# Hypoxia-inducible factor 2α is a negative regulator of osteoblastogenesis and bone mass accrual

**DOI:** 10.1038/s41413-019-0045-z

**Published:** 2019-02-21

**Authors:** Christophe Merceron, Kavitha Ranganathan, Elizabeth Wang, Zachary Tata, Shreya Makkapati, Mohd Parvez Khan, Laura Mangiavini, Angela Qing Yao, Laura Castellini, Benjamin Levi, Amato J. Giaccia, Ernestina Schipani

**Affiliations:** 1 Department of Orthopaedic Surgery, School of Medicine, University of Michigan, Ann Arbor, MI USA; 2 Division of Plastic and Reconstructive Surgery, Department of Surgery, University of Michigan, Ann Arbor, MI USA; 3 Department of Radiation Oncology, Stanford University Medical School, Stanford, CA USA

**Keywords:** Bone, Homeostasis

## Abstract

Osteoblasts, which are the bone-forming cells, operate in a hypoxic environment. The transcription factors hypoxia-inducible factor-1α (HIF1) and HIF2 are key mediators of the cellular response to hypoxia. Both are expressed in osteoblasts. HIF1 is known to be a positive regulator of bone formation. Conversely, the role of HIF2 in the control osteoblast biology is still poorly understood. In this study, we used mouse genetics to demonstrate that HIF2 is an inhibitor of osteoblastogenesis and bone mass accrual. Moreover, we provided evidence that HIF2 impairs osteoblast differentiation at least in part, by upregulating the transcription factor Sox9. Our findings constitute a paradigm shift, as activation of the hypoxia-signaling pathway has traditionally been associated with increased bone formation through HIF1. Inhibiting HIF2 could thus represent a therapeutic approach for the treatment of the low bone mass observed in chronic diseases, osteoporosis, or aging.

## Bone formation: Signaling in the opposite direction

The regulation of gene expression by hypoxia-inducible factor 2 (HIF2) prevents bone formation by inhibiting the differentiation of mesenchymal cells into osteoblasts. Ernestina Schipani at the University of Michigan, USA, and colleagues found that the bone mass of mice expressing a stabilized version of HIF2 was reduced compared with controls. This effect was associated with HIF2−mediated activation of the transcription factor SOX9, which disrupts osteoblastogenesis and stimulates the formation of cartilage cells. Interestingly, HIF1, which shares 48% overall amino acid identity with HIF2, is an established positive regulator of bone formation. These findings suggest that the opposing actions of HIF1 and HIF2 could be key for regulating bone growth. Furthermore, they raise the interesting possibility of using HIF2 inhibitors for the treatment of diseases characterized by low bone mass such as osteoporosis.

## Introduction

In the developing and adult skeleton, osteoblasts are the bone-forming cells and they originate from mesenchymal progenitors present in the periosteum and bone marrow.^1,2^ Notably, these mesenchymal progenitors can also give origin to chondrocytes in vitro when cultured in appropriate conditions.^3^ Two transcription factors, Runx2 and its downstream target, Sp7, have a crucial role in committing mesenchymal cells to become osteoprogenitors and thus osteoblasts, whereas the transcription factor Sox9 is required for differentiation of mesenchymal cells into chondrocytes.^4,5^ Osteoprogenitors proliferate and differentiate into postmitotic osteoblasts that synthesize and mineralize bone matrix; ultimately, osteoblasts either become inactive, give origin to osteocytes, which are cells embedded in the mineralized bone matrix,^6^ or die.^1^ Activity of the osteoblasts is coupled to activity of the osteoclasts, which are cells of hematopoietic origin whose role is to resorb bone. The osteoblast–osteoclast coupling allows for bone growth during development, and maintenance of bone and mineral homeostasis in adulthood.^7^

The bone marrow mesenchymal progenitors, also known as bone marrow stromal cells (BMSCs), and the osteoblasts lining the inner surface of trabecular and cortical bone exist and operate in a low-oxygen (hypoxic) environment.^8^ The transcription factors hypoxia-inducible factor-1α (HIF1) and HIF2 are critical mediators of the cellular response to hypoxia.^9^ They are both degraded by the proteasome in normoxic conditions, whereas they are stabilized by hypoxia, and thus their activity increases in hypoxic cells.^9^ By regulating a variety of biological functions, HIF1 and HIF2 allow cells to survive, proliferate, and differentiate in hypoxia.^9^ Depending on the cell type, these two transcription factors have been shown to have overlapping, unique, and even opposing actions.^10^ Notably, they are both expressed in cells of the osteoblast lineage.^8,11,12^ Analyses of mouse models carrying a gain-of-function or a loss-of-function mutation of osteoblastic HIF1 have established that HIF1 is a positive regulator of bone formation, as well as osteoblast number and activity in vivo, at least in part by stimulating non-oxidative glycolysis.^11,13^ Conversely, the role of HIF2 in the control of bone mass and osteoblast biology is still poorly understood.

In previous studies, it was reported that a loss-of-function mutation of HIF2 in osteoprogenitors or osteoblasts did not cause any significant change in bone mass or osteoblast number and activity.^8,11^ Interestingly, however, expression of a gain-of-function mutation of HIF2 in osteoprogenitors increased trabecular bone mass by impairing trabecular bone resorption.^8^ Furthermore, it caused polycythemia by stimulating erythropoietin (EPO) production and secretion by cells of the osteoblast lineage.^8,12^ Neither impairment of bone resorption nor polycythemia occurred upon expression of a gain-of-function mutation of HIF1 in the same cells.^8^ These findings suggest that HIF2 may have a unique and distinct role in osteoblastic cells when compared with HIF1.

In this study, we utilized mouse genetics to establish whether HIF2 regulates differentiation of mesenchymal progenitors into osteoblasts and bone mass accrual.

## Results

### In vitro constitutive stabilization of HIF2 inhibits differentiation of BMSCs into osteoblasts

Since low-oxygen tension stabilizes the HIF2 protein and bone in a hypoxic environment, we asked whether and how a stabilized osteoblastic HIF2 protein affects osteoblast differentiation. For this purpose, we took advantage of the HIF2dPA^f/f^ mice, in which a human cDNA encoding the mutant HIF2dPA protein is knocked into the ROSA26 locus and preceded by a stop cassette flanked by LoxP sites.^14^ HIF2dPA mutant protein cannot be hydroxylated by the oxygen sensors HIF prolyl-4-hydroxylases (PHDs) 1/2/3, because the two prolines that are the targets of the PHDs are mutated to alanines. Therefore, the HIF2dPA mutant protein escapes proteasomal degradation resulting in constitutive activation of HIF2 signaling regardless of oxygen tension.^14^ Transcriptional properties of this mutant protein are indistinguishable from those of wild-type HIF2.^14^

We isolated BMSCs from HIF2dPA^f/f^ mice by flushing of 6-week-old bones. Cells were cultured and transduced with adenovirus encoding *β-galactosidase* (Ad-LacZ, control) or *Cre recombinase* (Ad-Cre, mutant).^15^ Detection of alkaline phosphatase and Alizarin Red S staining were performed at 7 and 21 days, respectively, as previously described.^16,17^ 2-LoxP qPCR of genomic DNA revealed that the floxed allele was properly recombined in mutant cells with an efficiency of ~80% at both time points (data not shown).^14^

**Fig. 1:**
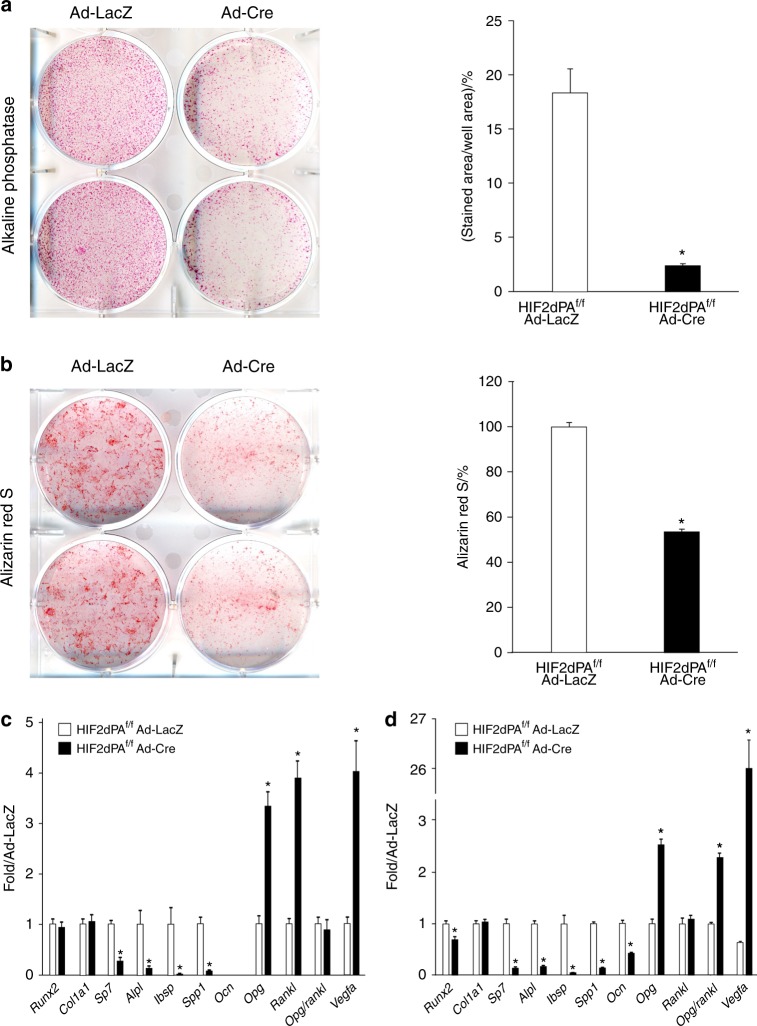
Expression of a gain-of-function mutation of HIF2 in BMSCs inhibits osteoblastogenesis in vitro. **a** ALP staining and **b** Alizarin Red S staining of BMSCs isolated from HIF2dPA^f/f^ mice, transduced with Ad-LacZ or Ad-Cre and cultured in osteogenic medium for 7 and 21 days, respectively. Representative images are shown on the left, and quantification of all biological replicates on the right. **c**, **d** qRT-PCR of total RNA extracted from the same BMSC cultures at d7 (**c**) and d21 (**d**). mRNAs encoding osteoblast markers (*Runx2, Col1a1, Sp7, Alpl, Ibsp, Spp1, Ocn, Opg, Rankl, and Opg/Rankl ratio*), and known downstream targets of HIF2 (*Vegfa* and *Opg*) are shown. Note that *Ocn* mRNA was virtually undetectable in both mutant and control cultures at d7, which is consistent with the early stage of osteoblastic differentiation of BMSCs at this time point. Data were normalized to expression of *TATA-Box Binding Protein* (*TBP*) mRNA. *P < 0.05

The number of alkaline phosphatase-positive cells was significantly lower in mutant cultures than in controls (Fig. 1a). Alizarin Red S staining, which detects the accumulation of calcium ions in mineralized tissues,^16^ was also dramatically decreased in mutants (Fig. 1b).

Consistent with the increased HIF2 activity, qRT-PCR of total RNA extracted from d7 and d21 BMSC mutant cultures showed that expression of the mRNA for vascular endothelial growth factor-A (*Vegfa*), which is a downstream target of HIF2,^18^ was increased to more than 4- and 25-fold at d7 and d21, respectively, when compared with control (Fig. 1c, d). Likewise, expression of osteoprotegerin mRNA (*Opg*) was significantly higher in mutant cells than in controls, which confirmed that *Opg* is most likely another direct downstream target of HIF2.^8^ Opg is a decoy receptor for receptor activator of nuclear factor kappa-Β ligand (*Rankl)* that is secreted by osteoblastic cells and inhibits osteoblast-dependent osteoclastogenesis.^8^ In mutant BMSCs, *Opg* mRNA expression was increased both at d7 and d21, whereas *Rankl* mRNA was only augmented at d7 that translates into a higher *Opg/Rankl* mRNA ratio in mature osteoblasts (Fig. 1c, d). The finding is consistent with the notion that overexpression of osteoblastic HIF2 inhibits bone resorption by increasing the Opg/Rankl ratio.^8^

More importantly, mRNAs for markers of osteoblast differentiation, including *Sp7*, alkaline phosphatase (*Alpl*), integrin-binding bone sialoprotein (*Ibsp*), osteopontin (*Spp1*), and osteocalcin (Ocn), were all severely downregulated in mutant cultures (Fig. 1c, d). *Sp7*, *Alpl, Ibsp*, and *Spp1* mRNAs are expressed starting from the early stages of the osteoblast differentiation process, whereas *Ocn* mRNA is produced by fully differentiated osteoblasts.^12,19,20^ However, levels of *Runx2* and type I collagen (*Col1a1*) mRNAs were comparable in mutants and controls (Fig. 1c, d). Last, mutant cells gained the ability to produce high amounts of *type II collagen* mRNA (*Col2a1*) at d7 and d21 and *aggrecan* (*Acan*) mRNA at d7. *Col2a1* and *Acan* are classical markers of chondrocytes (Supplementary Fig. 1).^21^

Our in vitro data demonstrate that constitutive stabilization of HIF2 does not affect the commitment of mesenchymal progenitors into osteoprogenitors, as mutant cells express normal amounts of *Runx2* and *Col1a1* mRNAs, but it impairs the differentiation of osteoprogenitors into osteoblasts. Moreover, it promotes expression of chondrogenic markers even in progenitor cells cultured in a monolayer.

### Constitutive stabilization of HIF2 in mesenchymal progenitors and their descendants in vivo inhibits bone formation

**Fig. 2:**
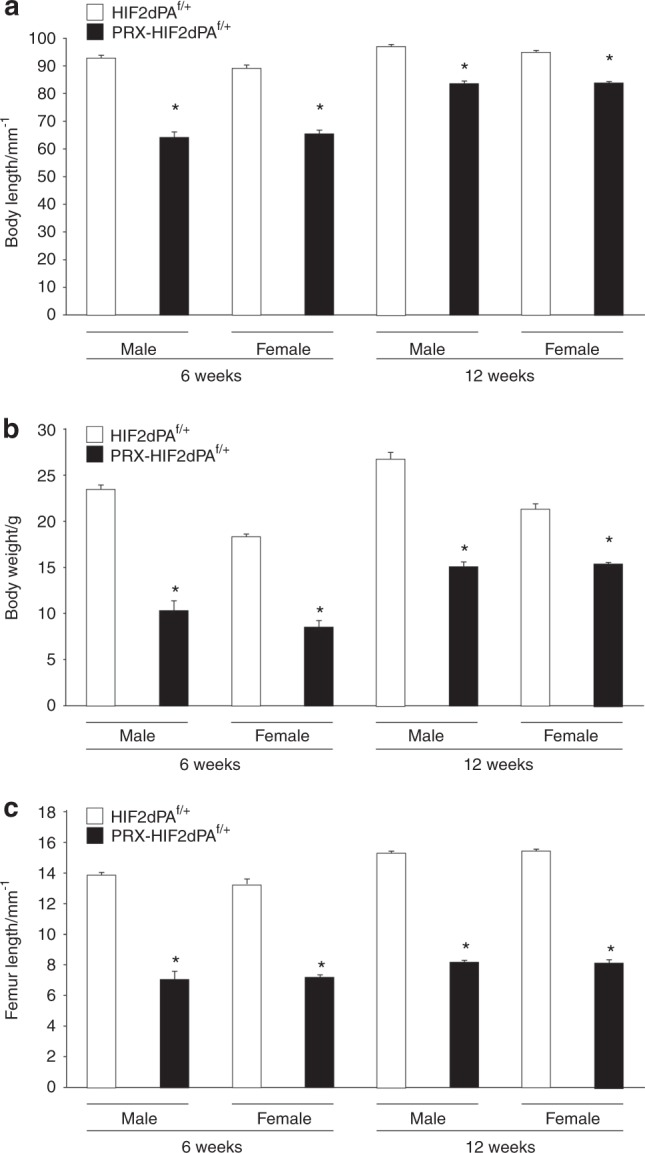
PRX-HIF2dPA^f/+^ mutant mice are smaller and lighter than controls. **a** Body length of male and female HIF2dPA^f/+^ control and PRX-HIF2dPA^f/+^ mutant mice at 6- and 12 weeks of age. **b** Weight of male and female HIF2dPA^f/+^ control and PRX-HIF2dPA^f/+^ mutant mice at the same time points. **c** Length of male and female HIF2dPA^f/+^ control and PRX-HIF2dPA^f/+^ mutant femurs at the same time points. *P < 0.05 referred to the comparison of HIF2dPA^f/f^ and PRX-HIF2dPA^f/+^ specimens matched for age and gender

Next, to determine whether our in vitro data could be relevant in vivo, we constitutively stabilized HIF2 in osteoblast precursors and their descendants by using the PRX1-Cre driver. For this purpose, we crossed PRX1-Cre transgenic mice to HIF2dPA^f/f^ mice.^22^ In PRX1-Cre, *Cre recombinase* is expressed in cells that give origin to the growth plate chondrocytes and the osteoblasts of the long bones, calvaria, and sternum.^22,23^ Heterozygous mutant mice (PRX-HIF2dPA^f/+^) were viable, and displayed a severe bone phenotype when compared with control mice (HIF2dPA^f/+^); therefore, generation of homozygous mutants was not pursued. Both males and females were analyzed at 6- and 12 weeks of age. Mutant mice were significantly smaller and lighter than controls and had shorter limbs, although no patterning defect could be identified (Fig. 2; data not shown). Since in PRX1-Cre transgenic mice, *Cre recombinase* is not expressed in the mesenchymal precursors of the vertebral bodies, it is likely that the shortening of the body in mutants as well as their reduced weight are the result of a yet unidentified systemic factor. Conversely, the reduced length of the limbs is, at least in part, secondary to abnormal growth plate development as histological analysis of developing growth plates isolated from newborn HIF2dPA^f/+^ and PRX-HIF2dPA^f/+^ mice showed a delay of chondrocyte hypertrophy in mutant specimens (Supplementary Fig. 2).

Consistent with our previous findings, circulating erythropoietin (EPO), hematocrit, and hemoglobin were all higher in PRX-HIF2dPA^f/+^ mutants when compared with controls (Supplementary Fig. 3).^8^

**Fig. 3:**
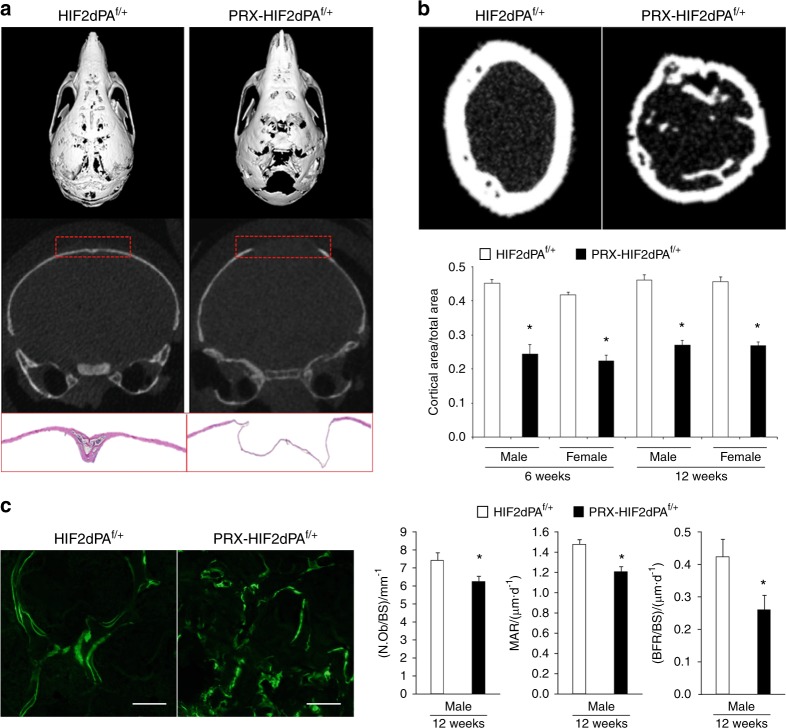
Expression of a gain-of-function mutation of HIF2 in osteoblast precursors inhibits bone formation and osteoblastogenesis in vivo. **a** At the top, micro-CT images of p21 HIF2dPA^f/+^ control and PRX-HIF2dPA^f/+^mutant skulls. Both the entire skull and coronal sections of the parietal bones are shown. Representative images are shown. Note the “hole” in the vault of the mutant skull, which makes the base of the skull visible. At the bottom, H&E staining of p21 HIF2dPA^f/+^ and PRX-HIF2dPA^f/+^parietal bones. **b** Micro-CT analysis of cortical bone of HIF2dPA^f/+^ and PRX-HIF2dPA^f/+^ femurs at 6- and 12 weeks of age. At the top, representative images of cross-sectional images of mid-diaphysis femurs are shown. At the bottom, quantification of cortical area/total area ratio for all biological replicates is provided. **c** Histomorphometric analysis of distal femur metaphyses of HIF2dPA^f/+^ and PRX-HIF2dPA^f/+^mice at 12 weeks of age. On the left, representative double-calcein labeling images are shown; on the right, quantification of N.Ob/BS, MAR, and BFR/BS is provided. *P < 0.05 referred to the comparison of HIF2dPA^f/+^ and PRX-HIF2dPA^f/+^ specimens matched for age and gender. Scale bars = 100 μm

PRX-HIF2dPA^f/+^ showed a dramatic delay of the osteogenic front in calvaria, which was clearly detectable at p21 and persisted at least up to 4 months of age (Fig. 3a; data not shown). Moreover, micro-CT analysis of PRX-HIF2dPA^f/+^ femurs revealed a severe thinning of the cortical bone at both 6- and 12 weeks of age and in both sexes (Fig. 3b). Last, the number of osteoblasts over bone surface (N.Ob/BS), the mineral apposition rate (MAR), and the bone-formation rate over bone surface (BFR/BS) were all significantly decreased in 12-week-old male mutants (Fig. 3c). These data indicate that both the osteoblast activity and the number of active osteoblasts at any given time was decreased in the trabecular compartment of mutant specimens.

**Fig. 4:**
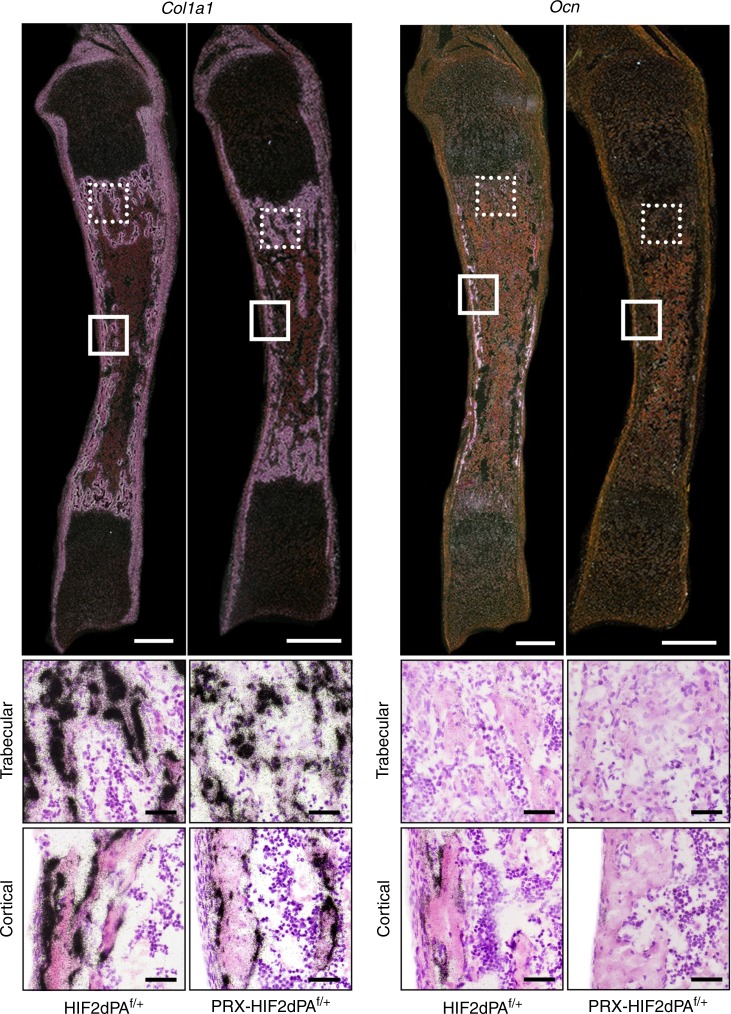
Expression of a gain-of-function mutation of HIF2 in osteoblast precursors inhibits their terminal differentiation in vivo. At the top, dark-field images of in situ hybridization for *Col1a1* (**a**) and *Ocn* (**b**) mRNAs of histological sections of HIF2dPA^f/+^ and PRX-HIF2dPA^f/+^ newborn tibias are shown. Scale bar = 1 mm. At the bottom, bright-field close-up images are provided. Scale bars = 50 μm

In agreement with our in vitro data, in situ hybridization analysis of histological sections of newborn tibias showed indistinguishable levels of *Col1a1* mRNA in the trabecular and cortical bone of both mutant and control (Fig. 4a). Conversely, *Ocn* mRNA was expressed by endosteal osteoblasts in control bones, whereas it was virtually undetectable in mutant osteoblasts at the same location, which indicates an impairment of osteoblast differentiation (Fig. 4b). Of note, consistent with previous publications, Ocn mRNA was not expressed by trabecular osteoblasts in either mutants or controls (Fig. 4b).^19^ Our in situ hybridization data support the notion that expression of a constitutively stabilized HIF2 in mesenchymal progenitors does not affect their commitment toward the osteoblast lineage, but it rather inhibits their differentiation into fully mature osteoblasts.

Taken together, the delay of the osteogenic front in the mutant skull, the thinning of the cortical bone, and the impaired bone-formation rate in the trabecular compartment of the long bones were all consistent with the HIF2-dependent impairment of osteoblastogenesis we had observed in vitro.

**Fig. 5:**
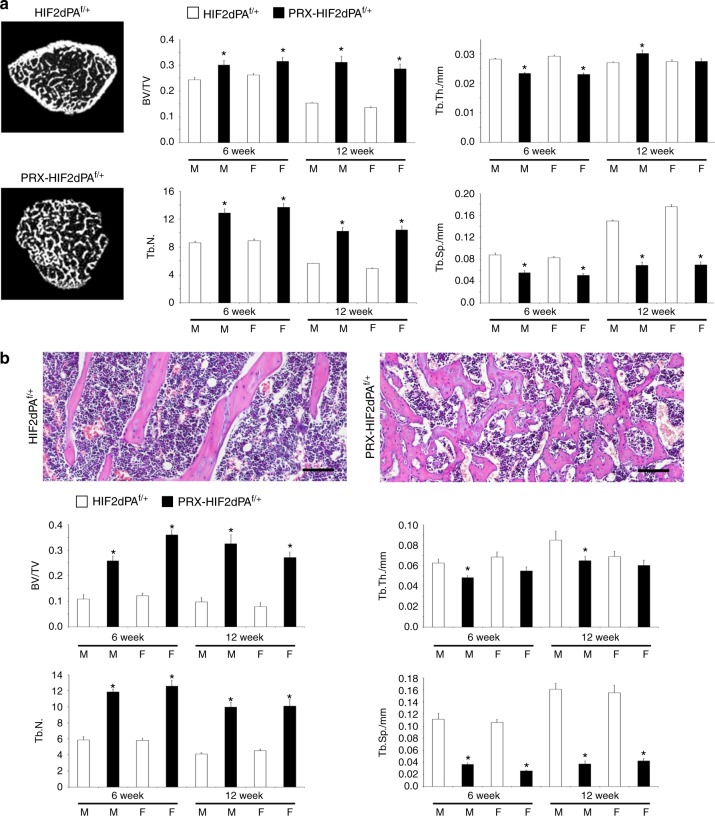
Trabecular bone mass is increased in PRX-HIF2dPA^f/+^ mutant mice. **a** Micro-CT analysis of distal femur metaphyses isolated from 6- and 12-week-old male and female HIF2dPA^f/+^ and PRX-HIF2dPA^f/+^ mice. Representative cross-section images are shown on the left; quantification of trabecular bone volume/tissue volume (BV/TV), trabecular thickness (Tb.Th.), trabecular number (Tb.N.), and trabecular separation (Tb.Sp.) is provided on the right. Note the virtual absence of cortical bone in the mutant metaphysis. **b** Histomorphometric analysis of distal femur metaphyses isolated from 6- and 12-week-old male and female HIF2dPA^f/+^ and PRX-HIF2dPA^f/+^ mice. At the top, representative paraffin sections stained with H&E are shown. Note the unique reticular pattern of the trabeculae and the presence of dilated blood vessels in mutant bones. The latter finding is consistent with the upregulation of Vegfa and inducible nitric oxide synthase (iNOS) detected in mutant cells (Fig. 1 and Fig. 9b). Quantification of the same parameters measured in (**a**) is provided at the bottom. M = male; F = female. *P < 0.05 referred to the comparison of HIF2dPA^f/f^ and PRX-HIF2dPA^f/+^ specimens matched for age and gender. Scale bars = 100 μm

Despite the impairment of bone formation, trabecular bone mass was increased in mutants (Fig. 5a, b), which in all probability was the result of a severe inhibition of bone resorption at this site, as documented by the persistence of cartilage remnants in the bone marrow of 6-week-old mutant bones (Supplementary Fig. 4A). Likewise, quantification of the number of osteoclasts over bone surface (N.Oc/BS) showed that osteoclast number was reduced in PRX-HIF2dPA^f/+^ when compared with controls (Supplementary Fig. 4B). These findings are in agreement with the inhibition of trabecular bone resorption by osteoblastic HIF2 we had previously reported.^8^

### In vivo loss of HIF2 in mesenchymal progenitors and their descendants augments bone-formation rate and leads to a high bone mass phenotype in both trabecular and cortical bone

**Fig. 6:**
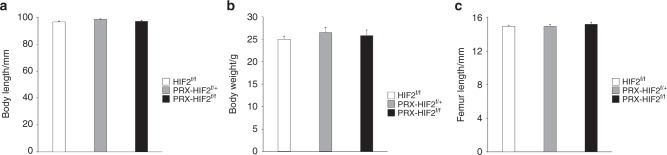
PRX-HIF2^f/f^ mutant mice are phenotypically similar to controls. **a** Body length and **b** body weight of male HIF2^f/f^, PRX-HIF2^f/+^, and PRX-HIF2^f/f^ mice at 12 weeks of age. **c** Length of 12-week-old male HIF2^f/f^, PRX-HIF2^f/+^, and PRX-HIF2^f/^ mice femurs

Next, we conditionally deleted HIF2 in mesenchymal progenitors and analyzed their bone phenotype to unveil the physiological functions of HIF2 in osteoblastic cells and establish whether HIF2 is necessary for the control of bone mass accrual. Since the gain-of-function experiment did not show any difference across genders and age, only males were investigated at 12 weeks of age. For this purpose, we crossed PRX1-Cre transgenic mice with HIF2^f/f^ mice to generate PRX-HIF2^f/f^ mutant and PRX-HIF2^f/+^ and HIF2^f/f^ control littermates.^24,25^ Of note, we had previously established that PRX1-HIF2^f/f^ mutants are viable and, prenatally, display only a modest and transient growth plate phenotype, which fully resolves postnatally.^24^ 2-LoxP qPCR of genomic DNA extracted from HIF2^f/f^ and PRX-HIF2^f/f^ BMSCs upon a brief in vitro culture showed that the floxed alleles had been efficiently recombined in mutant cells (efficiency of deletion 80% ± 0.02 SEM). Moreover, body weight, body length, and femur length of PRX-HIF2^f/f^ mutant mice were virtually indistinguishable from those of PRX-HIF2^f/+^ and HIF2^f/f^ control littermates (Fig. 6a–c). Last, hematocrit, hemoglobin, and circulating levels of EPO were similar across mutants and controls (Supplementary Fig. 5). This latter finding indicates that either HIF2 is not necessary for the regulation of osteoblastic EPO or osteoblastic EPO is not physiologically relevant as a source of serum EPO. The finding is consistent with the notion that kidney is the major source of EPO in physiological conditions.^12^

**Fig. 7:**
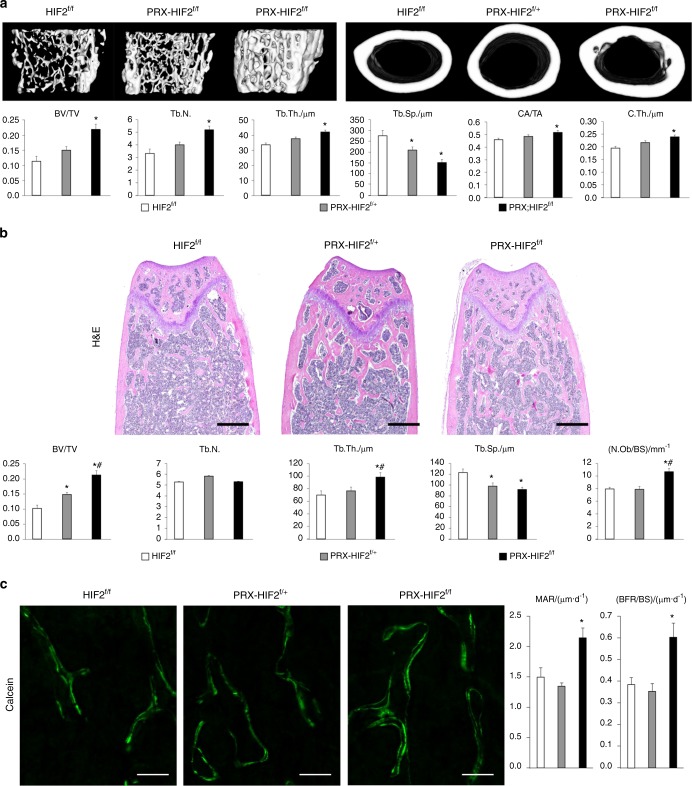
Loss of HIF2 in osteoblast precursors increases both trabecular and cortical bone mass. **a** Micro-CT analysis of trabecular bone in distal metaphysis and cortical bone in mid-diaphysis of femurs isolated from 12-week-old male HIF2^f/f^, PRX-HIF2^f/+^, and PRX-HIF2^f/f^ mice. At the top, representative images of trabecular (on the left) and cortical bone (on the right) are shown. At the bottom, quantification of BV/TV, Tb.N., Tb.Th., Tb.Sp. CA/TA, and Ct.Th. is provided. **b** Static histomorphometry analysis of trabecular bone of distal femurs isolated from 12-week-old male HIF2^f/f^, PRX-HIF2^f/+^, and PRX-HIF2^f/f^ mice. At the top, H&E staining of representative histological sections is shown. At the bottom, quantification of BV/TV, Tb.N., Tb.Th., Tb.Sp., and Ob.N./BS is provided. **c** Dynamic histomorphometric analysis of distal femurs isolated from 12-week-old male HIF2^f/f^, PRX-HIF2^f/+^, and PRX-HIF2^f/f^ mice. On the left, representative double-calcein labeling images are shown; on the right, quantification of MAR and BFR/BS is provided. Notably, a modest augmentation of some micro-CT and static histomophometric parameters was also noted in PRX-HIF2^f/+^ specimens, although in most cases, it did not reach statistical significance. *P < 0.05 referred to the comparison of HIF2^f/f^ and PRX-HIF2^f/f^ mice

Micro-CT analysis of femurs revealed a significant increase in both the trabecular and cortical bone mass of mutants when compared with controls (Fig. 7a–c). In particular, the trabecular bone volume over tissue volume (BV/TV), trabecular number (Tb.N.), and trabecular thickness (Tb.Th.) were all significantly higher in PRX-HIF2^f/f^ bones than in HIF2^f/f^ controls, where trabecular separation (Tb.Sp.) was significantly lower in mutants (Fig. 7a). In addition, cortical parameters, such as cortical area/total area (CA/TA) and cortical thickness (C.Th.), were also significantly increased in mutant specimens (Fig. 7a). Static histomorphometry analysis of trabecular bone confirmed the micro-CT data (Fig. 7b).

Furthermore, the number of osteoblasts over bone surface (N.Ob/BS), the mineral apposition rate (MAR), and the bone-formation rate over bone surface (BFR/BS) were all significantly augmented in mutant bones (Fig. 7b, c).

**Fig. 8:**
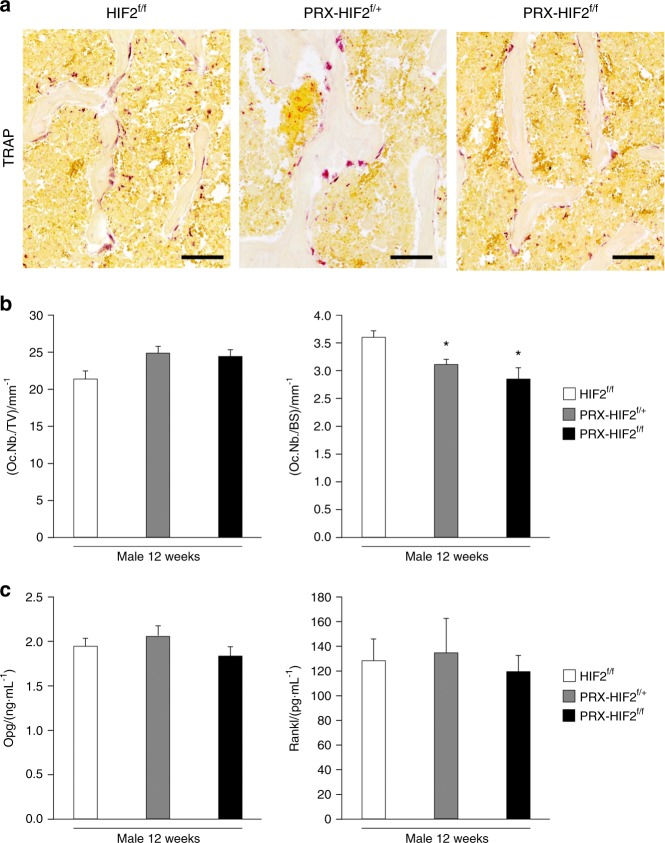
Osteoclast number is decreased in PRX-HIF2^f/f^ mutant bones. **a** TRAP staining of histological sections of distal epiphysis of femurs isolated from male HIF2^f/f^, PRX-HIF2^f/+^, and PRX-HIF2^f/f^ mice at 12 weeks of age. Representative images are shown. Scale bars = 100 μm. **b** Quantification of number of osteoclasts over tissue volume (Oc.Nb/TV) and number of osteoclasts over bone surface (Oc.Nb/BS) of all biological replicates is provided. **c** Measurements of circulating EPO in male HIF2^f/+^ PRX-HIF2^f/+^ and PRX-HIF2^f/f^ mutant mice at 12 weeks of age. *P < 0.05

Last, the osteoclast number over tissue volume (Oc.Nb./TV) was not different between HIF2^f/f^, PRX-HIF2^f/+^, and PRX-HIF2^f/f^ specimens. Conversely, the osteoclast number over bone surface (Oc.Nb./BS) was modestly reduced in PRX-HIF2^f/f^ bones in comparison with HIF2^f/f^ controls as a result of the increased bone surface in PRX-HIF2^f/f^ specimens secondary to the higher bone-formation rates in mutants (Fig. 8). In agreement with these data, measurements of circulating Opg and Rankl did not show any significant difference between mutants and controls (Fig. 8). Therefore, HIF2 is not necessary for osteoclastogenesis to occur, although its increased activity is sufficient to inhibit osteoclast formation.

Taken together, our in vivo findings indicate that HIF2 has a physiological and non-redundant role as a negative regulator of bone mass in both trabecular and cortical bone, mainly by decreasing bone formation.

Next, we complemented the in vivo data with an in vitro assay in order to prove that HIF2 controls osteoblast differentiation through a direct action on osteoblast precursors. For this purpose, BMSCs were isolated from HIF2^f/f^ bones, transduced with Ad-LacZ (control cells) or Ad-Cre (mutant cells), and cultured in 20% O_2_ (normoxia) or 1% O_2_ (hypoxia) for 21 days. Of note, 20% O_2_ and 1% O_2_ are both arbitrary choices. Since bone and bone marrow are vascularized tissues, it is highly likely that mesenchymal progenitors and osteoblastic cells in vivo are exposed to cyclical changes in O_2_ levels rather than steady-state hypoxia. 2-LoxP qPCR of genomic DNA revealed that the HIF2 floxed allele was properly recombined in mutant cells with an efficiency of ~80% (Supplementary Fig. 6A). Moreover, HIF2 mRNA was virtually undetectable in mutant cells (Supplementary Fig. 6A). Last, the hypoxia-dependent increase of Vegfa was significantly reduced upon loss of HIF2. Of note, Vegfa is a direct downstream target of both HIF2 and HIF1.^26^

Alizarin Red S staining revealed a dramatic impairment of mineralization in both control and mutant cells cultured in hypoxia (data not shown). This finding indicates that prolonged hypoxia impairs osteoblast differentiation and/or mineralization with mechanisms that are HIF2-independent^27^ and may be secondary to other modalities, including changes of pH, as exposure to hypoxia is known to lead to a progressive acidification of the extracellular medium in a HIF1-dependent manner.^28^ Anyhow, the data do not disprove the hypothesis that HIF2 is a negative regulator of osteoblastogenesis.

More importantly, loss of HIF2 significantly increased Alizarin Red S staining of normoxic cultures (Supplementary Fig. 7), which confirmed our in vivo data and showed that HIF2 is an inhibitor of osteoblast differentiation independently of the degree of oxygenation. The finding is consistent with the hypothesis that HIF2 is a negative regulator of osteoblastogenesis. Of note, cell number was not different between mutant and control cultures (data not shown).

It has been recently reported that HIF1 is a positive regulator of bone formation. Therefore, in principle, the increased bone mass and the augmented bone-formation rates observed in PRX-HIF2^f/f^ mutant mice could be secondary to a compensatory increase of osteoblastic HIF1. To explore this possibility, we analyzed HIF1 accumulation and activity in the same mutant BMSCs lacking HIF2 described above. As expected, exposure to hypoxia increased HIF1 protein accumulation and activity without modifying its mRNA levels in control cultures (Supplementary Fig. 8). More importantly, loss of HIF2 did not further increase the levels of HIF1 mRNA, protein, or activity (Supplementary Fig. 8). Taken together, our findings show that it is highly unlikely that the bone phenotype in PRX-HIF2^f/f^ mutant mice is mediated by a compensatory increase of osteoblastic HIF1.

Next, to further prove in vivo that HIF1 is not required for the increase in bone mass observed upon loss of osteoblastic HIF2, we generated PRX-HIF1^f/f^–HIF2^f/f^ double-mutant mice. Unfortunately, PRX-HIF1^f/f^–HIF2^f/f^ mutant growth plates displayed a massive cell death phenotype (Supplementary Fig. 9; data not shown) that was indistinguishable from the one previously reported in PRX-HIF1^f/f^ single-mutant mice. The severe chondrocyte death and the extreme deformities of PRX-HIF1^f/f^–HIF2^f/f^ mutant limbs (data not shown) precluded any meaningful analysis of their adult bone phenotype.

### Sox9 is a downstream target of HIF2 in osteoblastic cells

HIF2 is known as a transcriptional activator rather than a transcriptional repressor. Therefore, it is unlikely that HIF2 impairs osteoblast differentiation by directly inhibiting transcription of osteoblastic genes.

**Fig. 9:**
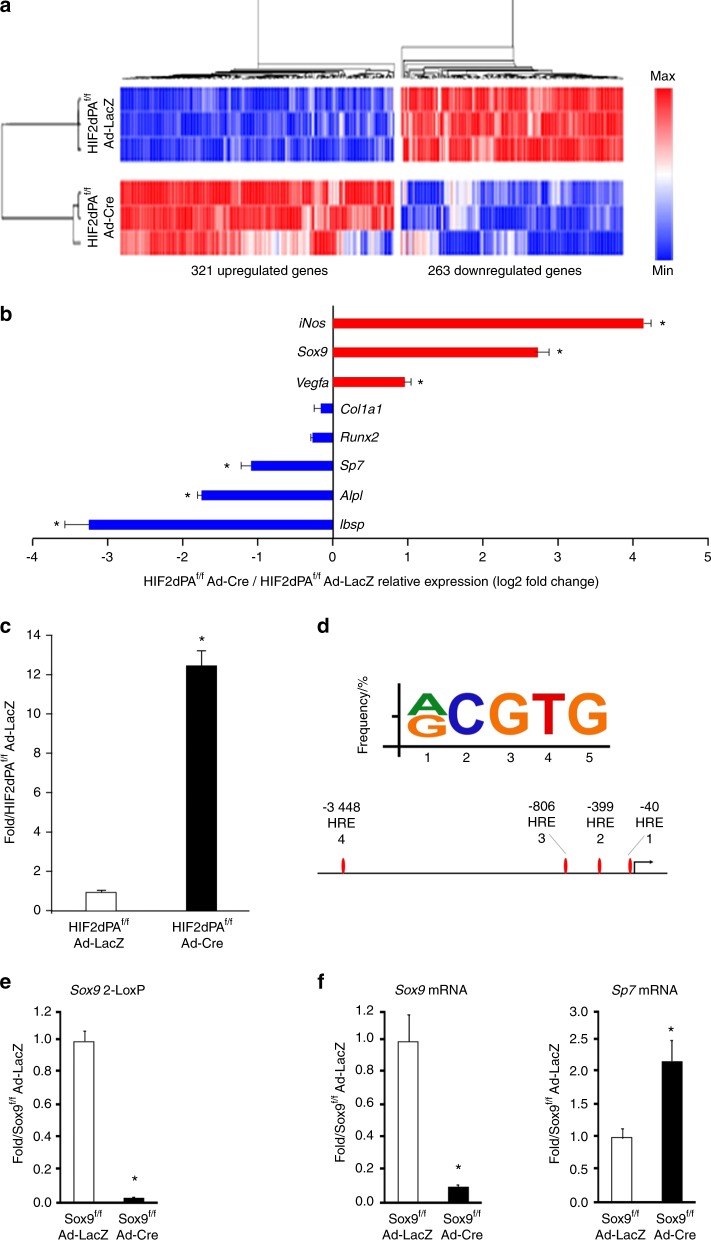
Sox9 is a downstream target of HIF2 in BMSCs. **a** mRNA microarray analysis of genes regulated by the HIF2dPA mutant protein in BMSCs. Hierarchical representation of gene cluster distribution depending on gene expression levels. **b** Selected number of upregulated (red) and downregulated (blue) genes. **c** qRT-PCR of total RNA isolated from HIF2adPA^f/f^ BMSCs and transduced with Ad-LacZ or Ad-Cre. *Sox9* mRNA is shown. Data were normalized to expression of *TBP* mRNA. **d** Bioinformatics analysis of the HRE motif in the Sox9 promoter. Consensus sequence (5′-[A/G]CGTG-3′) of HRE and JASPAR predicted HREs within –3 500/ + 100-bp region of the mouse Sox9 promoter are shown. **e** 2-LoxP qPCR of genomic DNA isolated from Sox9^f/f^ BMSCs and transduced with Ad-LacZ or Ad-Cre. Data were normalized to Vhl as an internal control for genomic DNA input. **f** qRT-PCR of total RNA isolated from Sox9^f/f^ BMSCs and transduced with Ad-LacZ or Ad-Cre. *Sox9 and Sp7* mRNAs are shown. Data were normalized to expression of *TBP* mRNA. *P < 0.05

Microarray analysis of total RNA revealed that, in agreement with the results of the in vitro cell differentiation assays, expression of a constitutively stabilized HIF2dPA protein in BMSCs significantly decreased expression of numerous mRNAs encoding classical osteoblastic proteins, such as *Sp7*, *Ibsp*, and *Alpl* (Fig. 9a, b).

Notably, levels of *Runx2* and *Col1a1* mRNAs were unchanged in mutant cells when compared with controls (Fig. 9b), which confirms our qRT-PCR findings (Fig. 1c) and further indicates that the HIF2dPA mutation had not affected the commitment of BMSCs toward the osteoblast lineage.

More importantly, our microarray analysis showed that Sox9 mRNA was robustly expressed at baseline in BMSCs and was also the most upregulated mRNA, i.e., sevenfold over baseline, encoding a transcription factor upon expression of a constitutively stabilized HIF2 mutant in these cells. Microarray data were confirmed by qRT-PCR (Fig. 9c).

We thus searched the Sox9 promoter for hypoxia-responsive elements (HREs), the binding site of HIFs on DNA, containing a conserved RCGTG sequence by using bioinformatics tools. Potential HREs located within the Sox9 promoter were predicted using the JASPAR CORE database with a relative score threshold of 0.85. In agreement with previously published findings, our analysis revealed the presence of four HREs within the Sox9 promoter adjacent to the transcriptional start site (Fig. 9d).^29,30^ This finding suggests that Sox9 could be a direct transcriptional target of HIF2.

Recent studies have proposed that Sox9 is not only a master transcription factor of chondrogenesis, but it is also an inhibitor of osteoblastogenesis as it prevents chondrocytes from differentiating into osteoblasts, interferes with Runx2 function in committed osteoblastic cells, and inhibits BMP2-induced osteoblastogenesis.^31–33^ Notably, HIF2 is a known positive regulator of Sox9 expression in articular surface chondrocytes.^34^ It is thus possible to hypothesize that Sox9 could mediate the HIF2-dependent inhibition of osteoblast differentiation. In support of this hypothesis, genetic loss of Sox9 in BMSCs significantly increased expression of *Sp7* mRNA (Fig. 9e, f).

## Discussion

Our study shows that HIF2 is a negative regulator of bone mass accrual by impairing bone formation through a direct action on cells of the osteoblast lineage and with a molecular mechanism that may require osteoblastic Sox9. Our findings constitute a paradigm shift, as activation of the HIF signaling pathway through HIF1 has been associated with increased, rather than decreased, osteoblast activity. They thus suggest that, as in other cell types, HIF1 and HIF2 may have opposing functions in regulating osteoblast activity.^10^ Mechanistically, Sox9, which is emerging as a negative regulator of osteoblast differentiation, is likely to mediate, at least in part, the HIF2-dependent impairment of osteoblastogenesis.^31–33^

Establishing the exact interplay between HIF1 and HIF2 in cells of the osteoblast lineage is a crucial question that warrants further investigations. In particular, novel mouse models in which loss-of-function mutations of both HIF1 and HIF2 are temporally induced in mesenchymal progenitors and their descendants need to be generated and characterized. Those novel mouse models will allow the devastating effect of the loss of HIF1 on the growth plate development to be overcome.

In this paper, we have also documented that overexpression of HIF2 in mesenchymal progenitors of the limb bud impairs chondrocyte hypertrophy. However, it is unlikely that the bone phenotype displayed by PRX-HIF2dPA^f/+^ mutant mice is the consequence of their altered growth plate development. Both the impairment of osteoblast differentiation observed in vitro in progenitors expressing a gain-of-function mutation of HIF2 and the delay of the osteogenic front present in mutant PRX-HIF2dPA^f/+^calvaria, i.e., in bones that develop through an intramembranous process, support our conclusion. In any event, it is important to note that the bone phenotype observed in PRX-HIF2dPA^f/+^ mutants could be the result of either an alteration of bone modeling, remodeling, or both. Further studies are warranted to distinguish between these two possibilities.

The inhibition of bone formation and osteoblastogenesis in PRX-HIF2dPA^f/+^ mice occurred despite the increased production of *Vegfa* mRNA we observed in mutant osteoblasts in vitro. Overexpression of osteoblastic *Vegfa* augments osteoblast number and activity through a variety of mechanisms involving both paracrine and autocrine actions.^35^ Our findings suggest that constitutive stabilization of HIF2 in osteoblastic cells triggers a molecular mechanism that overrides Vegfa’s actions and leads to inhibition of bone formation and osteoblastogenesis.

Notably, our in vitro data revealed that HIF2 does not affect the commitment of mesenchymal progenitors into osteoblastic cells, but it impairs their subsequent differentiation by acting downstream of Runx2 and upstream of Sp7. They thus explain why, differently from PRX-HIF2dPA^f/+^ mutant mice, no overt bone phenotype could be observed in mice lacking HIF2 in either Sp7 or Ocn lineage cells.^8,11^

Mechanistically, the notion that Sox9 mediates, at least in part, the HIF2-dependent inhibition of osteoblastogenesis is in agreement with previous data showing that Sox9 interferes with Runx2 function in committed osteoblastic cells.^32^

Mutant HIF2dPA BMSCs produce markers of the chondrocyte lineage in vitro, which suggests that HIF2 not only inhibits osteoblast differentiation but somehow promotes chondrogenesis, at least in vitro. The role of HIF2 in promoting chondrogenic differentiation of undifferentiated progenitors warrants further investigations.

More relevant to our study, no ectopic chondrocytes could be detected in the mutant HIF2dPA bone marrow in vivo (data not shown). The data are also consistent with the absence of cartilage in the normal bone marrow, even if a gradient of oxygen is indeed present in bone and HIFs are stabilized in normal osteoblastic cells.^8,12,35,36^ Furthermore, they suggest that activation of HIF2 alone is not sufficient to support the full chondrogenic program in the bone marrow in vivo.

Loss of HIF2 in osteoblast precursors increases bone mass in both trabecular and cortical bone. HIF2 can be selectively inhibited by small molecules,^37^ some of which are currently in clinical trials in patients carrying pathologies associated with high levels of HIF2 activity, such as clear cell renal carcinoma.^37^ Therefore, HIF2 could become a novel target for the treatment of low bone mass seen in chronic diseases, osteoporosis, and with aging. Studies aimed at determining whether the HIF2-dependent regulation of bone mass also occurs in the adult bone, independently of its putative action during development, are warranted.

## Materials and methods

### Animals and generation of PRX;HIF2dPA^f/+^ and PRX;HIF2^f/f^ mutant mice

Generation and genotyping methods of Prx1-Cre (FVB/N), HIF2dPA^f/f^ (C57BL/6), HIF1^f/f^ (FVB/N), HIF2^f/f^ (C57BL/6), and Sox9^f/f^ (C57BL/6 J) transgenic mice have been previously described.^14,22,25,38^ Primers used for genotyping are provided in Table 1.

**Table 1:** Genotyping and 2-LoxP primers

Primers	Sequence	Amplicon/bp
*HIF2dPA*	Fwd 5′-TTGCCCCCTCTTCCCCTCGTGATCT-3′	300
	Rev 5′-CCGGTAGAATTCCTGCAGGTCGAGGG-3′	
*HIF1*	Fwd 5′-TGATGTGGGTGCTGGTGTC-3′	350
	Rev 5′-TTGTGTTGGGGCAGTACTG-3′	
*HIF2*	Fwd 5′-CAGGCAGTATGCCTGGCTAATTCCAGTT-3′	460
	Rev 5′-CTTCTTCCATCATCTGGGATCTGGGACT-3′	
*Sox9*	Fwd 5′-CCGGCTGCTGGGAAAGTATATG-3′	350
	Rev 5′-CGGTGGTATTCAGGGAGGTACA-3′	
*Vhl*	Fwd 5′-CTAGGCACCGAGCTTAGAGGTTTGCG-3′	310
	Rev 5′-CTGACTTCCACTGATGCTTGTCACAG-3′	

PRX1-Cre transgenic mice were crossed with HIF2dPA^f/f^ mice. Heterozygous mutant mice (PRX-HIF2dPA^f/+^) were viable, and displayed a severe bone phenotype when compared with control mice (HIF2dPA^f/+^); therefore, generation of homozygous mutants was not pursued.

Prx1-Cre male mice were bred with homozygous HIF2^f/f^ females, in order to obtain PRX-HIF2^f/+^ male mice. These newly generated males were crossed with HIF2^f/f^ females to generate PRX-HIF2^f/f^ mutants and PRX-HIF2^f/+^ and HIF2^f/f^ control littermates.

HIF1^f/f^–HIF2^f/f^ female mice were generated and crossed with Prx1-Cre male to obtain PRX-HIF1^f/+^–HIF2^f/+^ male mice. These newly generated males were crossed with HIF1^f/f^–HIF2^f/f^ females to generate PRX-HIF1^f/f^–HIF2^f/f^ mutants and PRX-HIF1^f/+^–HIF2^f/+^ and HIF1^f/f^–HIF2^f/f^ control littermates.

All procedures involving mice were performed in accordance with the NIH guidelines for use and care of live animals and were approved by the University of Michigan Institutional Animal Care and Use Committee (IACUC) (Protocol number: PRO00007215).

### BMSC isolation and cell culture

HIF2dPA^f/f^, HIF2^f/f^, or Sox9^f/f^ bone marrow stromal cells (BMSCs) were obtained by flushing of 6-week-old bones, cultured in high-glucose DMEM supplemented with 15% FBS and 1% penicillin/streptomycin (growth medium), and incubated at 37 °C, 5% CO_2_ under a humidified atmosphere. The medium was renewed every 24 h during the first 48 h to remove nonadherent cells; the medium was next changed every 2–3 days. Cells were grown up to 90% confluence, and replated at a density of 40 × 10^3^ cells per cm^2^. For all experiments, BMSCs were used at passage 1. HIF2dPA^f/f^ or HIF2^f/f^ BMSCs were transduced with adenovirus encoding *β-galactosidase* (Ad-LacZ) or *Cre recombinase* (Ad-Cre) at a multiplicity of infection of 100 during 3 consecutive days.^15^

Efficiency of deletion was assessed by qPCR for 2-LoxP performed on genomic DNA at the indicated time.^14,25^ Data were normalized to *Von Hippel–Lindau* (*Vhl)* as internal reference for genomic DNA.

### Alkaline phosphatase (ALP) and Alizarin Red S stainings

Upon transduction, BMSCs were cultured in differentiation medium (growth medium supplemented with ascorbic acid (50 μg·mL^−1^) and β-glycerophosphate (10 mmol·L^−1^)) for the indicated time. The medium was changed every 2–3 days. HIF2dPA^f/f^ BMSCs were cultured at 37 °C, in normoxic conditions (20% O_2_, 5% CO_2_) under a humidified atmosphere. HIF2^f/f^ BMSCs were cultured either in 20% O_2_ (normoxia) or 1% O_2_ (hypoxia) (H35 hypoxystation, Don Whitley Scientific).

Detection of alkaline phosphatase activity and Alizarin Red S staining were performed at 7 and 21 days, respectively, as previously described.^15,17^ Briefly, ALP staining was performed according to the manufacturer’s instructions (Sigma-86C-1KT).

In regard to the Alizarin Red S staining, after fixation in 4% PFA, calcium–phosphate deposits were stained with Alizarin red S according to the manufacturer’s instructions (Sigma-Aldrich—A5533). Alizarin Red S dye was then eluted and quantified by colorimetric detection at 405 nm as described elsewhere.^16^

### Quantitative real-time PCR

Total RNA was extracted from BMSCs using RNA-Bee reagent (Tel-Test Inc.) according to the manufacturer’s protocol. Total RNA concentration was determined by Nanodrop. Reverse transcription of mRNA was performed using Omniscript Reverse Transcriptase (Qiagen) as the manufacturer’s protocol recommended. The *TATA-box binding protein gene* (*TBP*) mRNA was amplified as an internal control. Relative mRNA levels were calculated using the 2^−ΔΔCt^ method.^39^ Primer sequences are listed in Table 2.

**Table 2:** Primers used for qRT-PCR of total RNA

Primers	Sequence	Amplicon/bp
*Tbp*	Fwd 5′-AGAACAATCCAGACTAGCAGCA-3′	120
	Rev 5′-GGGAACTTCACATCACAGCTC-3′	
*HIF2*	Fwd 5′-CTGAGGAAGGAGAAATCCCGT-3′	161
	Rev 5′-TGTGTCCGAAGGAAGCTGATG-3′	
*Runx2*	Fwd 5′-TGTTCTCTGATCGCCTCAGTG-3′	146
	Rev 5′-CCTGGGATCTGTAATCTGACTCT-3′	
*Col1*	Fwd 5′-TGTGTGCGATGACGTGCAAT-3′	133
	Rev 5′-GGGTCCCTCGACTCCTACA-3′	
*Sp7*	Fwd 5′-GGAAAGGAGGCACAAAGAAGC-3′	218
	Rev 5′-CCCCTTAGGCACTAGGAGC-3′	
*Alpl*	Fwd 5′-CGGATCCTGACCAAAAACC-3′	74
	Rev 5′-TCATGATGTCCGTGGTCAAT-3′	
*Ibsp*	Fwd 5′-GAAAATGGAGACGGCGATAG-3′	73
	Rev 5′-CATTGTTTTCCTCTTCGTTTGA-3′	
*Spp1*	Fwd 5′-AGCAAGAAACTCTTCCAAGCAA-3′	134
	Rev 5′-GTGAGATTCGTCAGATTCATCCG-3′	
*Ocn*	Fwd 5′-AGACTCCGGCGCTACCTT-3′	85
	Rev 5′-AAGCAGGGTCAAGCTCACAT-3′	
*Opg*	Fwd 5′-GTTTCCCGAGGACCACAAT-3′	71
	Rev 5′-CCATTCAATGATGTCCAGGAG-3′	
*Rankl*	Fwd 5′-TGAAGACACACTACCTGACTCCTG-3′	84
	Rev 5′-CCCACAATGTGTTGCAGTTC-3′	
*Vegfa*	Fwd 5′-CTTGTTCAGAGCGGAGAAAGC-3′	125
	Rev 5′-ACATCTGCAAGTACGTTCGTT-3′	
*Col2a1*	Fwd 5′-ACTTGCCAAGACCTGAAACTCTG-3′	97
	Rev 5′-AAACTTTCATGGCGTCCAAGG-3′	
*Acan*	Fwd 5′-CCTGCTACTTCATCGACCCC-3′	150
	Rev 5′-AGATGCTGTTGACTCGAACCT-3′	
*Sox9*	Fwd 5′-AGTACCCGCATCTGCACAAC-3′	145
	Rev 5ʹ-TACTTGTAATCGGGGTGGTCT-3′	
*Glut1*	Fwd 5ʹ-CAGTTCGGCTATAACACTGGTG-3′	156
	Rev 5′-GCCCCCGACAGAGAAGATG-3′	
*Hif1a*	Fwd 5′-TCTCGGCGAAGCAAAGAGTC-3′	214
	Rev 5′-AGCCATCTAGGGCTTTCAGATAA-3′	

### Western blotting

Proteins were extracted in RIPA buffer in the presence of protease inhibitors (Roche). Protein concentration was estimated by the BCA assay (Thermo Scientific). For each sample, 20 μg of protein were resuspended in sample buffer and electrophoresed in a precast 4%–20% Tris gel (BioRad). After gel transfer to polyvinylidene fluoride (PVDF) membranes using a BioRad Criterion system, blots were blocked in 5% nonfat milk/1x TBST for 1 h at room temperature and incubated overnight at 4 °C with the following primary antibodies: Hif1a (Novus Biologicals: NB100479) at 1:1 000, and α-tubulin (Cell Signaling: #2125) at 1:10 000. The membranes were then incubated with a HRP-conjugated anti-rabbit IgG (Santa Cruz: sc-2004) at 1:2 000 for 1 h at room temperature in 5% no-fat milk/1x TBST. The signal was detected by using enhanced chemiluminescence (ECL Prime Western Blotting System GE Healthcare, RPN2232). Western blot images were acquired and analyzed via the BioRad Image Lab system. Quantification was performed using ImageJ. The α-tubulin signal was used to normalize for protein amount.

### Microarray analysis

Microarray analysis was performed using Affymetrix technology (Mouse Gene ST 2.1 Strip) on total RNA extracted from BMSCs isolated from 6-week-old HIF2dPA^f/f^ mice. These cells were transduced with Ad-LacZ or Ad-Cre for 3 consecutive days at a MOI of 1:100, and cultured in growth medium at 20% O_2_ for an additional 72 h after transduction. RNA was extracted using RNA-Bee reagent as described above. RNA integrity was assessed using the Eukaryote Total RNA pico kit in association with the Bioanalyzer 2100 Expert (Agilent). RNA integrity numbers (RIN) were over 9.6 for all of the samples. For each probe set, the expression level of the control group was compared with the Ad-Cre-treated group. Among the 26 500 coding transcripts, 584 referenced genes with a fold change (FC) in expression level ≥ 1.5 (log2 FC ≥ 0.58) and *P*-values < 0.05 were identified as significantly regulated. All these transcripts were also robustly expressed at baseline (lowest average expression value = 2.5).^40^

### Blood analysis

Blood was collected by cardiac puncture under deep anesthesia (surgical plane) using a 1-mL syringe fitted with a 23-G needle. Blood samples were split into two fractions. The first fraction was collected in K_2_EDTA-coated tubes (BD Microtainer—365974) for complete blood count (CBC). CBC was performed by the Unit for Laboratory Animal Medicine—*In Vivo* Animal Core (ULAM-IVAC) using a Hemavet 950FS (Drew Scientific). The other fractions were collected in serum separator tubes (BD Microtainer—365967). Blood was allowed to clot for 30 min at room temperature prior to centrifugation for 5 min at 10 000 g. Samples were stored at −80 °C prior to EPO, Opg, and Rankl quantification by ELISA according to the manufacturer’s instructions (RnD Systems—MEP00B, MOP00, and MTR00).^8,12^

### Micro-CT analysis

3D micro-CT images of the femora were generated using the eXplore Locus SP system (GE Healthcare). Images were analyzed and quantified using Microview Software (Parallax Innovations). Regions of interest (ROI) were defined as a percentage of the overall bone length. Both trabecular and cortical ROI lengths were set at 10% of the overall bone length. Trabecular ROI position was defined by having its closest edge located 5% away from the growth plate. Cortical ROI position was defined by having its center at 50% of the total bone length.

### Routine histology, H&E staining, Safranin-O staining, TRAP staining, and in situ hybridization analysis

For routine histology, H&E, Safranin-O, and tartrate-resistant acidic phosphatase (TRAP) stainings, tissue was fixed in 4% PFA/PBS and processed as previously reported.^41^ TRAP staining was performed according to the manufacturer’s indications (Sigma-Aldrich, 387A-1KT). In situ hybridization (ISH) analysis was performed using complementary ^35^S-labeled riboprobes as previously described.^41^ H&E-, Safranin-O-, and TRAP-stained sections were used for static histomorphometry analysis. All images were acquired with an Eclipse E800 microscope (Nikon).

### Histomorphometry

Histomorphometric analysis was performed on mice of 6- and 12 weeks of age matched for genotype and gender. ROI were defined as a percentage of the overall bone length. Both trabecular and cortical ROI lengths were set at 10% for the gain-of-function and 20% for the loss-of-function experiment of the overall bone length. Trabecular ROI position was defined by having its closest edge located at 3% for the gain-of-function and 5% for the loss-of-function experiment away from the growth plate. Cortical ROI position was defined by having its center at 50% of the total bone length. Histomorphometric measurements were performed in a randomized and blind manner using the Bioquant Osteo software V17.2.6 (Bioquant Image Analysis Corp., Nashville, TN), according to standard procedures.^42^

#### Static histomorphometry

H&E sections were used to quantify trabecular bone volume over tissue volume (BV/TV), bone surface (BS), trabecular number (Tb.N), trabecular thickness (Tb.Th.), trabecular spacing (Tb.Sp.), and number of osteoblast over bone surface (N.Ob/BS). Cartilage remnants quantification was performed on Safranin-O-stained sections. TRAP staining allowed the visualization and proper counting of the number of osteoclasts over tissue volume (Oc.Nb/TV) and number of osteoclasts over bone surface (Oc.Nb/BS).

#### Dynamic histomorphometry

Mice were injected intraperitoneally with calcein (Sigma-C0875) (40 mg·kg^−1^) 10 and 3 days prior to sacrifice (Sims JCI 2000). Femurs were collected and fixed in PFA 4% prior to embedding in methylmethacrylate as previously described.^19,20^ Sections of 10-μm thickness were cut and coverslipped without any staining. Observation and image capture for dynamic parameter measurements (mineral apposition rate (MAR) and bone formation rate over bone surface (BFR/BS) were performed using a FITC filter mounted on an Eclipse E800 microscope (Nikon).

### Bioinformatics analysis

The chromosomal location and the 3.5-kb promoter sequence of the murine *Sox9* gene were retrieved using the Ensembl! database (ENSMUSG00000000567). The presence of putative HRE (5′-[A/G]CGTG-3′) located within the promoter of the murine sox9 gene was assessed using the Jaspar core database (http://jaspar.genereg.net/) with a relative threshold of 0.85.^43^

### Statistical analysis

All the in vitro experiments listed above were performed at least in biological and technical triplicates. For an in vivo experiment, a minimum of six specimens per condition were analyzed. Data are represented as the mean of the replicates ± SEM. A two-tailed, unpaired Student’s *t* test was performed to analyze statistical differences between groups in all cases. *P*-values < 0.05 were considered statistically significant.

## Supplementary information

Supplementary figures

Supplementary Legend
