# Supplementary material for: Hypoxia-inducible factor 2α is a negative regulator of osteoblastogenesis and bone mass accrual
Source: Bone Res. 2019 Feb 21;7:7. doi: 10.1038/s41413-019-0045-z (PMC6382776; doi:10.1038/s41413-019-0045-z)
Supplement: Supplementary file 1 — Supplementary figures [file 41413_2019_45_MOESM1_ESM.pdf]

Supplementary Fig. 1

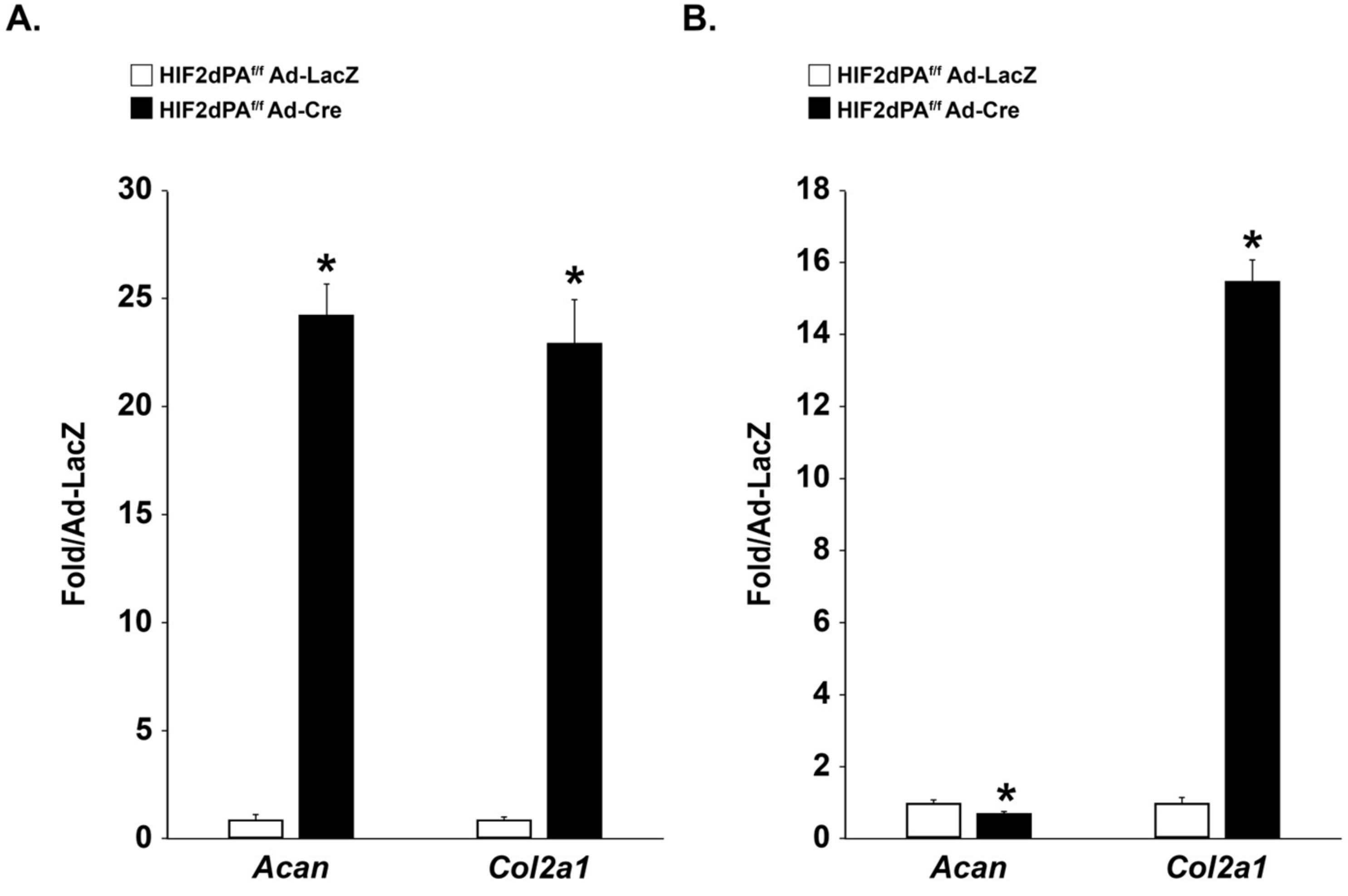

Supplementary Fig. 2

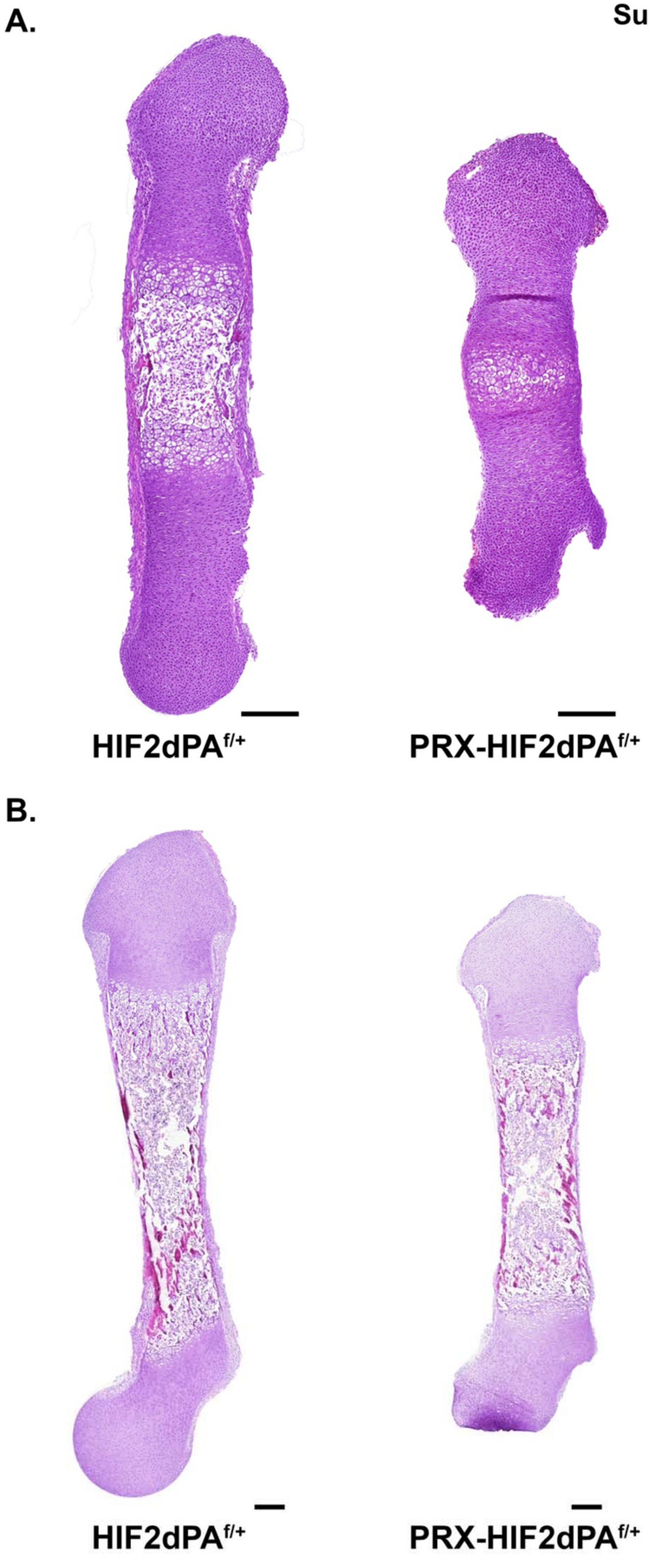

Supplementary Fig. 3

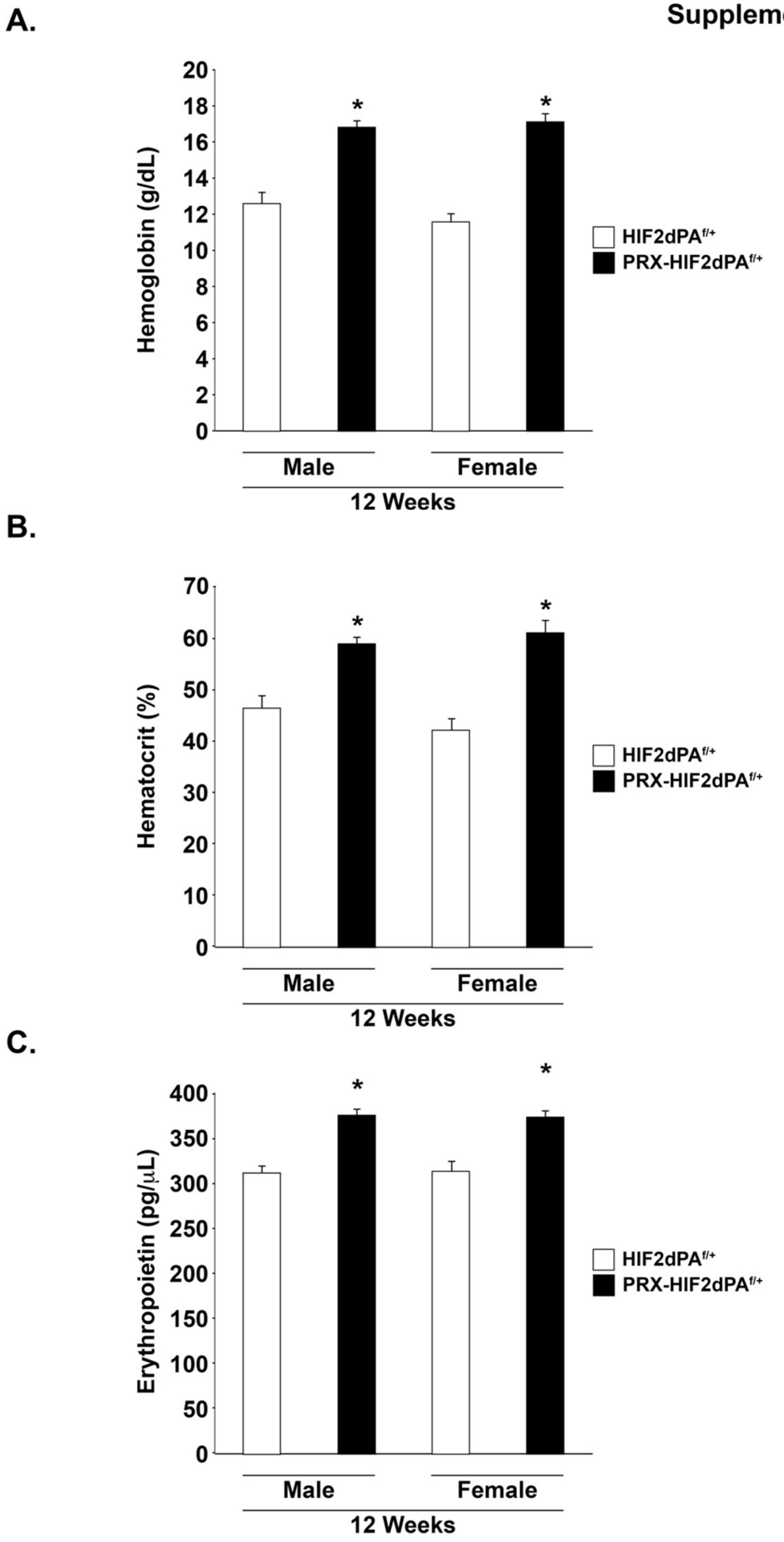

Supplementary Fig. 4

A.

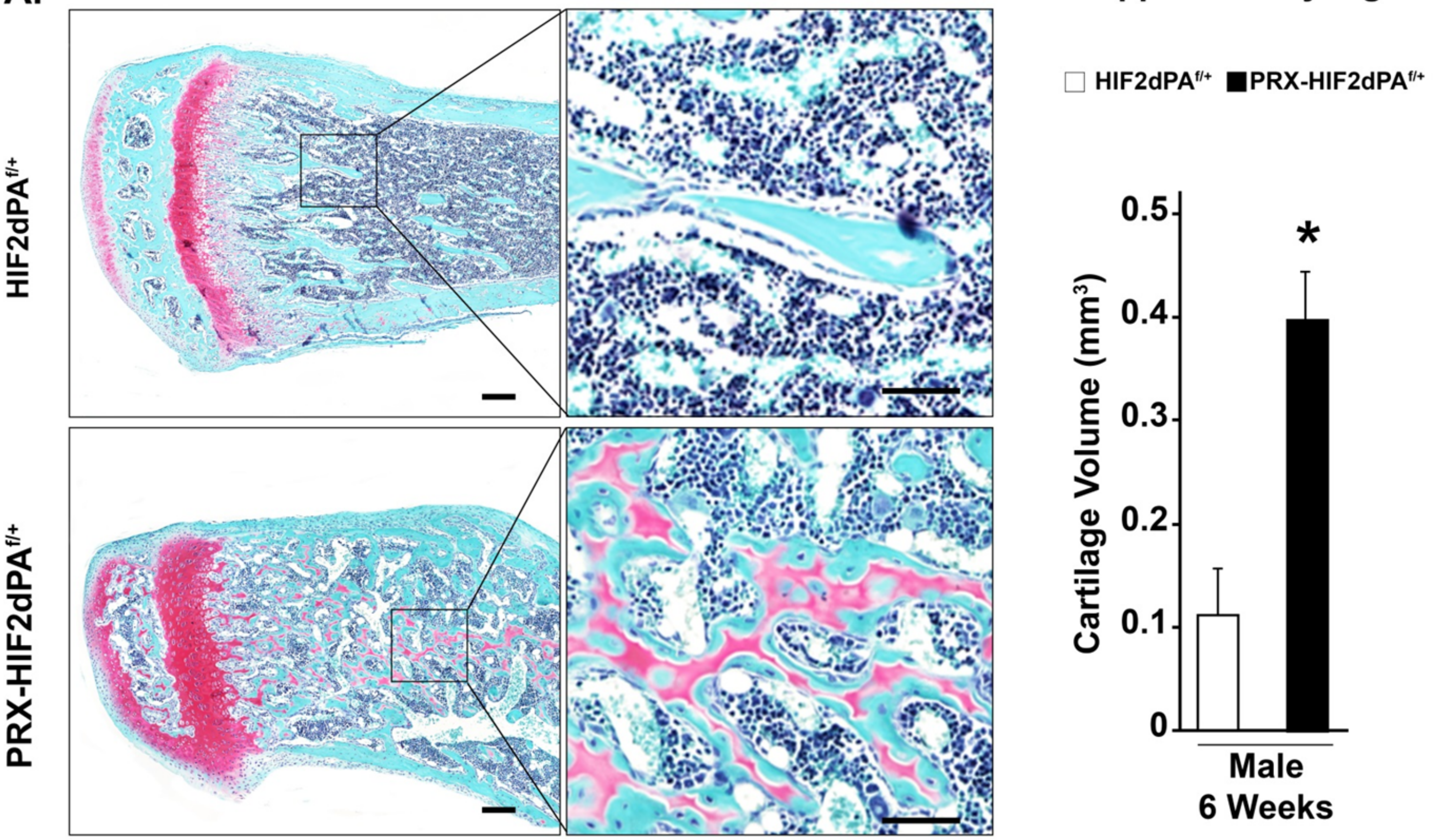

B.

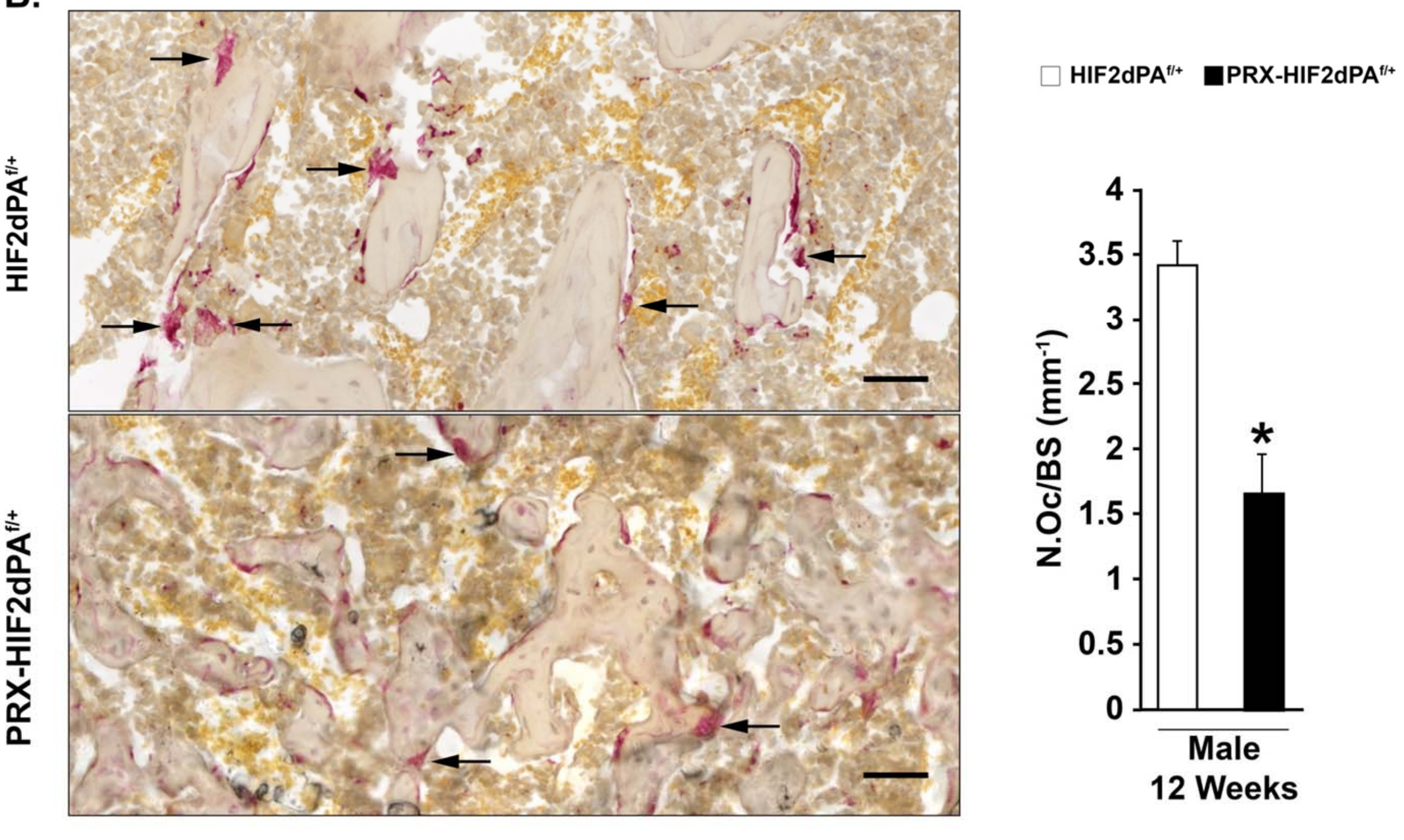

Supplementary Fig. 5

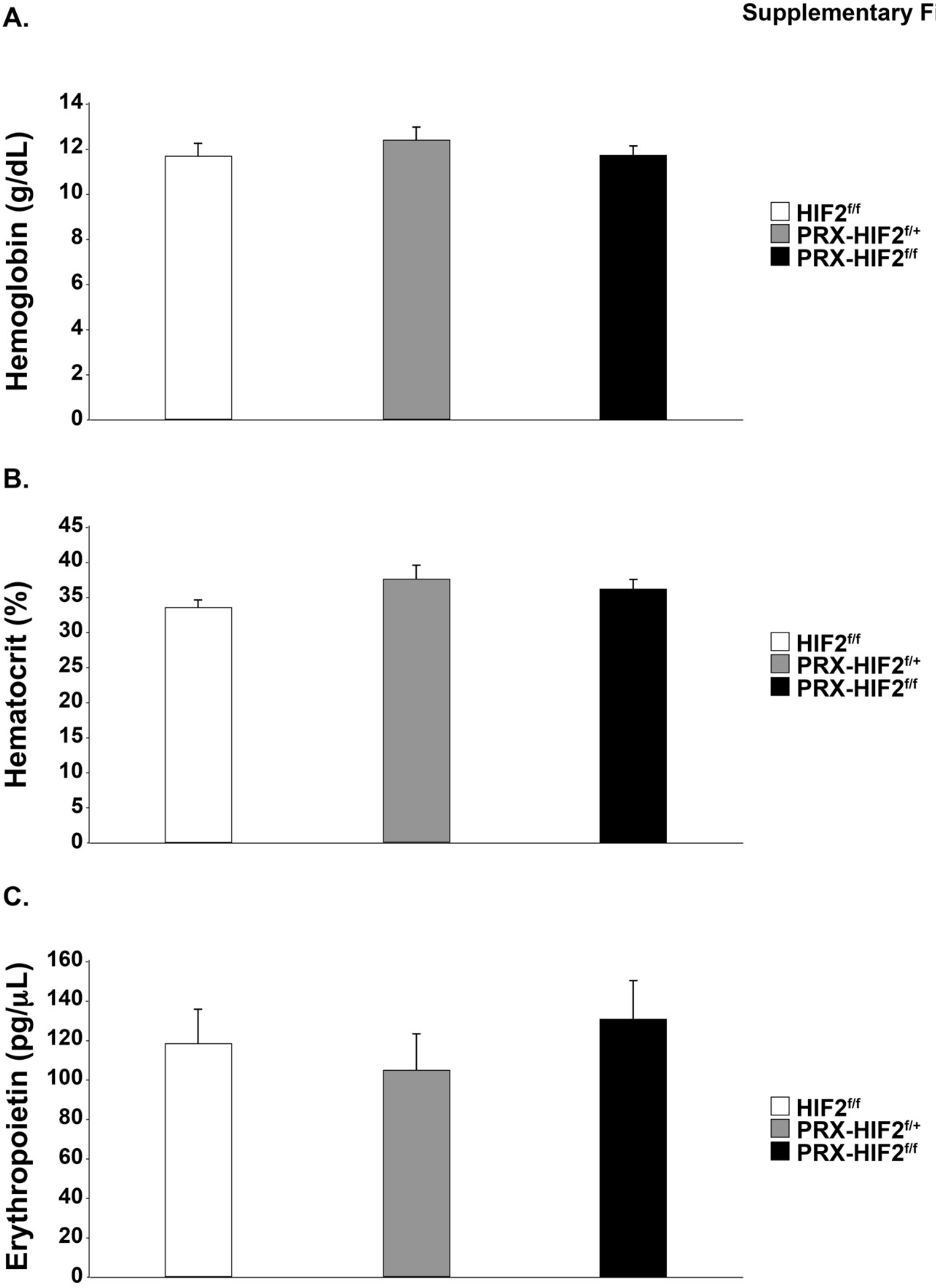

Supplementary Fig. 6

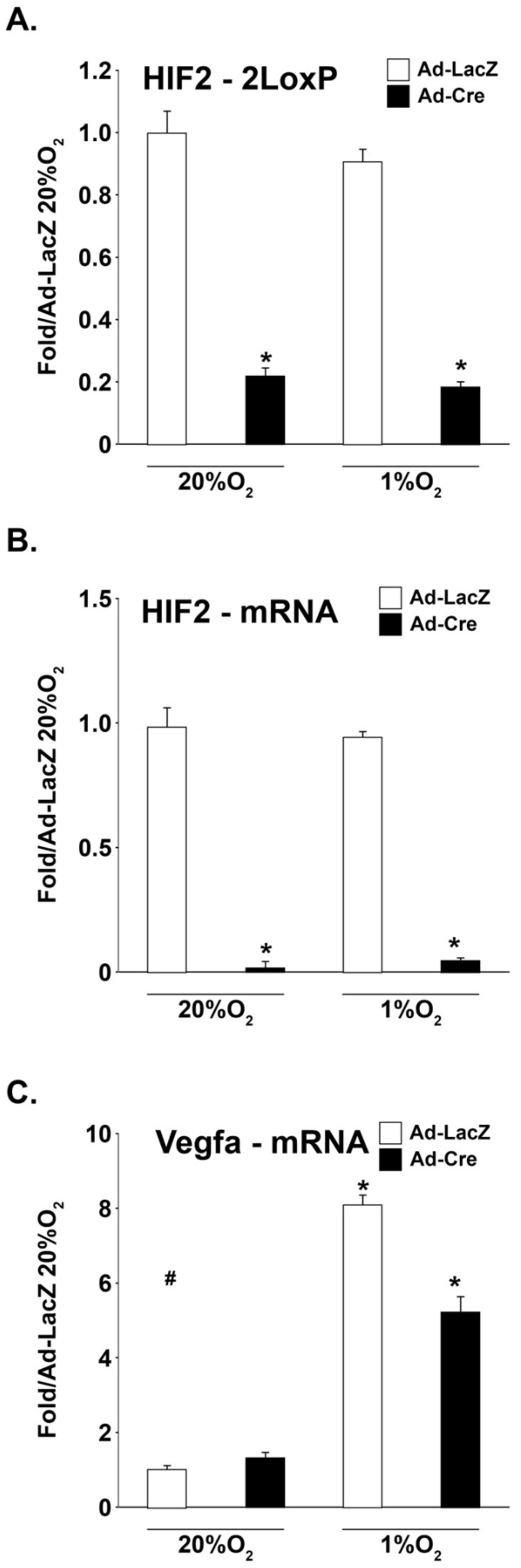

Supplementary Fig. 7

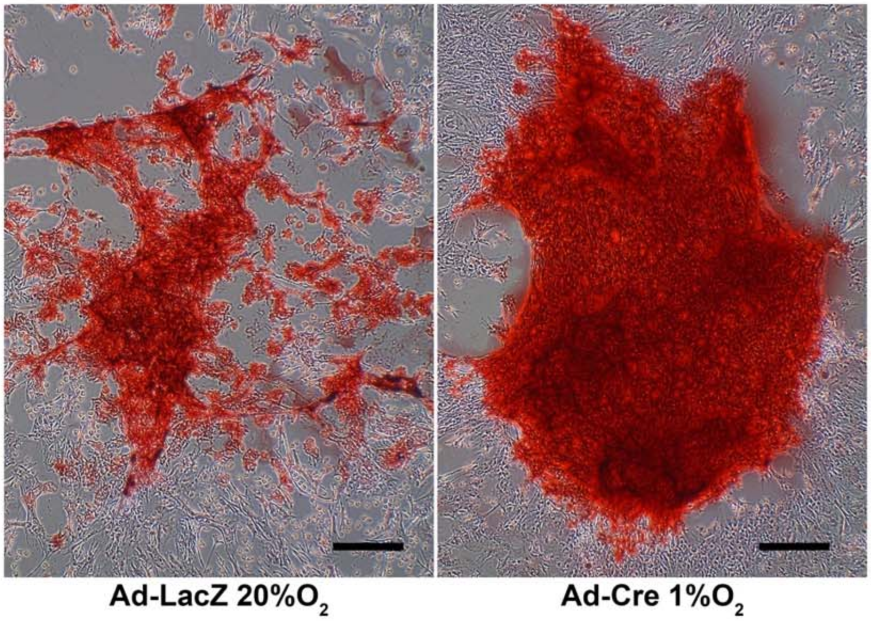

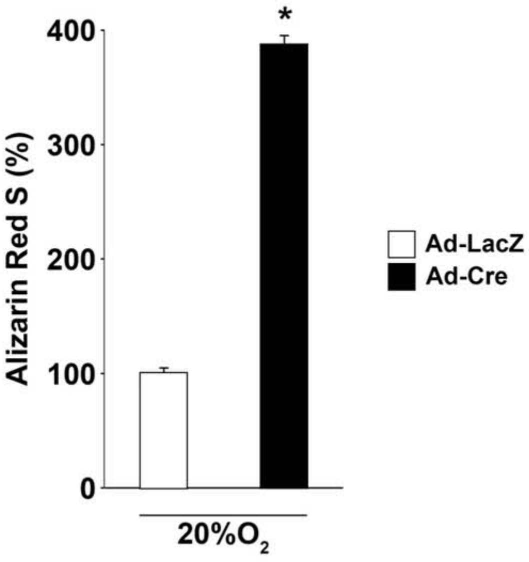

Supplementary Fig. 8

A.

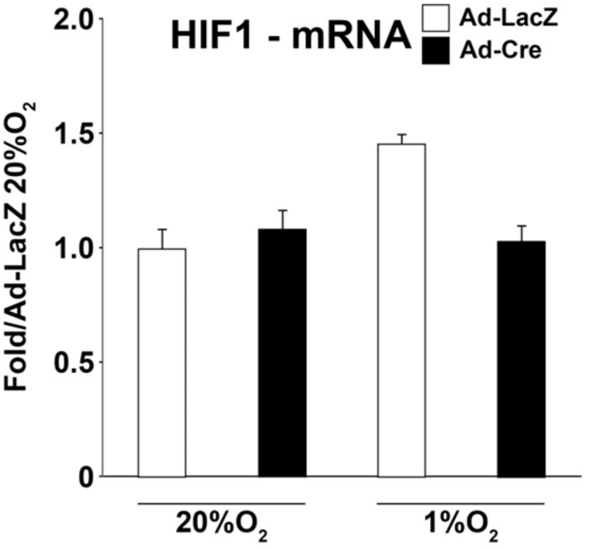

B.

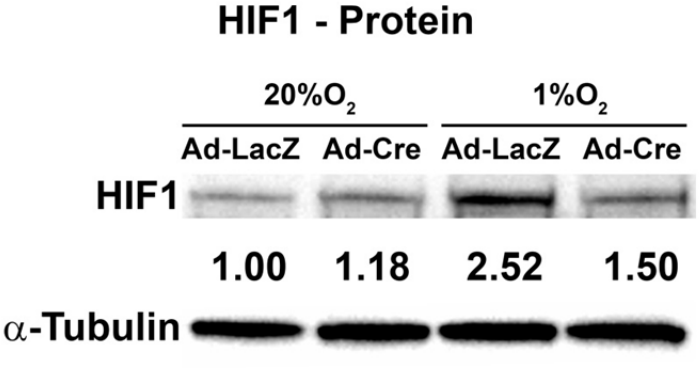

C.

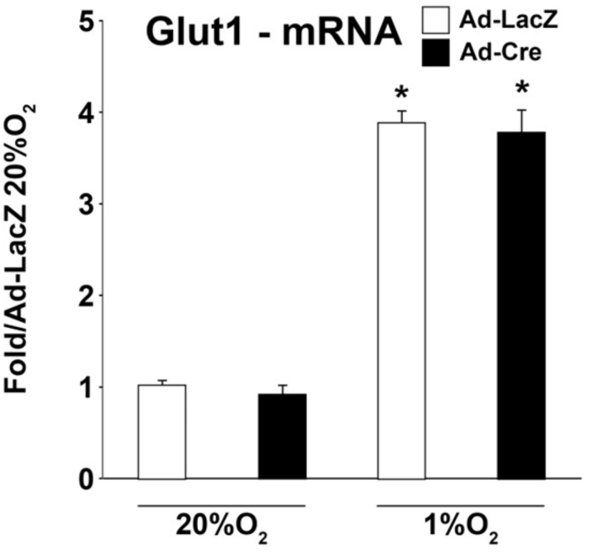

Supplementary Fig. 9

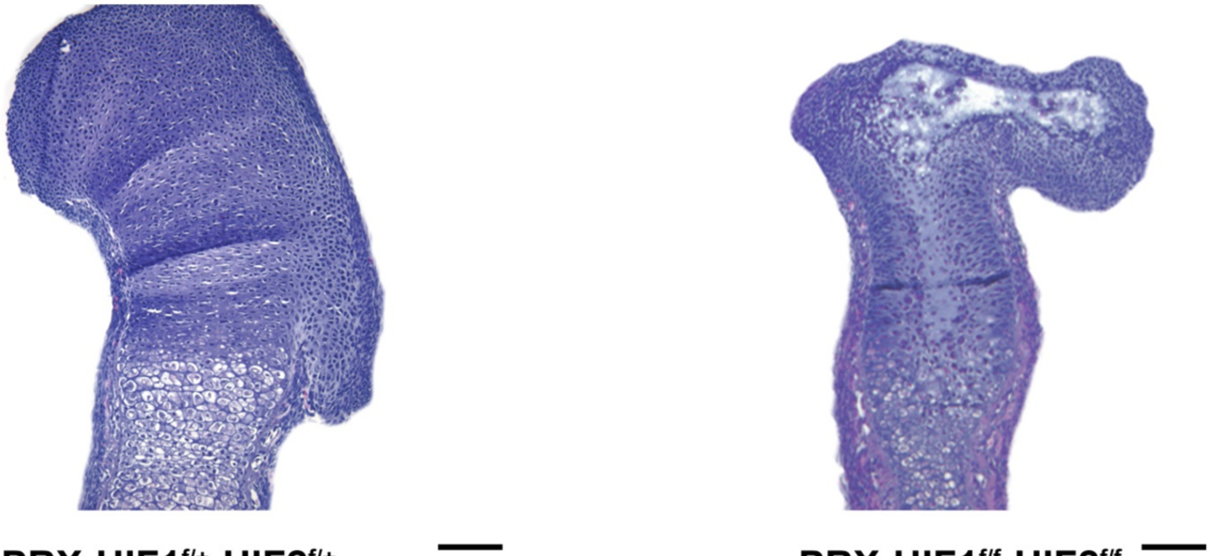

PRX-HIF1$^{f/+}$-HIF2$^{f/+}$

PRX-HIF1$^{f/f}$-HIF2$^{f/f}$
