# Supplementary material for: Hypoxia-inducible factor 2α is a negative regulator of osteoblastogenesis and bone mass accrual
Source: Bone Res. 2019 Feb 21;7:7. doi: 10.1038/s41413-019-0045-z (PMC6382776; doi:10.1038/s41413-019-0045-z)
Supplement: Supplementary file 2 — Supplementary Legend [file 41413_2019_45_MOESM2_ESM.docx]

**Supplementary figure 1: Expression of a gain-of-function mutation of HIF2 in BMSCs increases expression of chondrogenic markers *in vitro***. (A, B) qRT-PCR of total RNA extracted from BMSCs isolated from HIF2dPA^f/f^ mice, transduced with Ad-LacZ or Ad-Cre and cultured in osteogenic medium for 7 (A) and 21 days (B). mRNAs encoding chondrocyte markers (*Acan* and *Col2a1*) are shown. Data were normalized to expression of *TATA-Box Binding Protein* (*TBP*) mRNA. *p<0.05.

**Supplementary figure 2: Expression of a gain-of-function of HIF2 in mesenchymal progenitors of the limb bud delays chondrocyte hypertrophy.** Histological sections of femurs isolated from HIF2dPA^f/+^ control and PRX-HIF2dPA^f/+^ mutant mice at E15.5 (A) and birth (B). H&E staining is shown. Scale bars=200μm

**Supplementary figure 3: Hemoglobin, hematocrit and circulating levels of EPO are increased in PRX-HIF2dPA^f/+^ mutant mice.** Measurements of hemoglobin (A), hematocrit (B) and circulating EPO (C) in male and female HIF2dPA^f/+^ control and PRX-HIF2dPA^f/+^ mutant mice at 12-weeks of age. *p<0.05 referred to the comparison of HIF2dPA^f/+^ and PRX-HIF2dPA^f/+^ specimens matched for gender.

**Supplementary figure 4:** **Osteoclast number and activity is impaired in PRX-HIF2dPA^f/+^ mutant bones**. (A) Safranin-O staining of histological sections of 6-week-old male HIF2dPA^f/+^ and PRX-HIF2dPA^f/+^ tibias. Representative images are shown on the left; quantification of cartilage remnants is provided on the right. Scale bars=200μm; 25μm for close ups. (B) TRAP staining of histological sections of 12-week-old male HIF2dPA^f/+^ and PRX-HIF2dPA^f/+^ femurs. Representative images are shown on the left; arrows point to positively stained cells. Quantification of number of osteoclasts over bone surface (N.Oc/BS) is provided on the right. *p<0.05. Scale bars=100μm.

**Supplementary figure 5: Hematocrit and circulating levels of EPO are similar in PRX-HIF2^f/f^ mutant and control mice.** Measurements of hemoglobin (A), hematocrit (B) and circulating EPO (C) in male HIF2^f/f^, PRX-HIF2^f/+^ and PRX-HIF2^f/f^ mice at 12-weeks of age.

**Supplementary figure 6: The floxed HIF2 locus in BMSCs is efficiently recombined *in vitro*.** (A) 2-LoxP qPCR of genomic DNA extracted from BMSCs isolated from HIF2^f/f^ mice, transduced with Ad-LacZ or Ad-Cre and cultured in osteogenic medium for 21 days in either 20% or 1% O_2_. Data were normalized to *von Hippel Lindau* gene as internal control for genomic DNA. Quantification of *HIF2 (B) and* Vegfa (C) mRNAs, by qRT-PCR performed on total RNA extracted from the cells cultured as described above. Vegfa is a direct downstream target of both HIF2 and HIF1. Data were normalized to *TATA-box Binding Protein* (*TBP*).

**Supplementary figure 7: Loss of HIF2 promotes the *in vitro* mineralization of BMSCs in normoxia.** Alizarin red S staining of BMSCs isolated from HIF2^f/f^ mice transduced *in vitro* with Ad-LacZ or Ad-Cre and cultured in osteogenic medium for 21 days. Quantification is shown on the right. Scale bars=50μm. *p<0.05

**Supplementary figure 8: Recombination of HIF2, *in vitro*, does not increase HIF1 expression, accumulation or activity in BMSCs.**

Quantification of *HIF1(A) and glucose transporter 1 (Glut1) (C)* mRNAs by qRT-PCR performed on total RNA extracted from the cells cultured as described above. Data were normalized to *TATA-box Binding Protein* (*TBP*). Glut1 is a direct downstream target of HIF1 transcriptional activity (B) Quantification of HIF1 protein by Western blot analysis of total protein lysate extracted from BMSCs cultured as described above. A representative Western blot with quantification below is shown. Data were normalized to α-tubulin. *p<0.05 for samples compared to HIF2a^f/f^ Ad-LacZ 20%O_2_; #p<0.05 for samples compared to HIF2a^f/f^ Ad-LacZ 1%O_2_.

**Supplementary figure 9: Loss of HIF2 does not prevent the severe growth plate phenotype secondary to loss of HIF1.** H&E stained histological sections of humerus isolated from PRX-HIF1^f/+^-HIF2^f/+^ control and PRX-HIF1^f/f^-HIF2^f/f^ mutant mice E14.5.
